# Identification and targeting of a HES1‐YAP1‐CDKN1C functional interaction in fusion‐negative rhabdomyosarcoma

**DOI:** 10.1002/1878-0261.13304

**Published:** 2022-08-29

**Authors:** Alexander R. Kovach, Kristianne M. Oristian, David G. Kirsch, Rex C. Bentley, Changde Cheng, Xiang Chen, Po‐Han Chen, Jen‐Tsan Ashley Chi, Corinne M. Linardic

**Affiliations:** ^1^ Department of Pediatrics Duke University School of Medicine Durham NC USA; ^2^ Department of Pharmacology & Cancer Biology Duke University School of Medicine Durham NC USA; ^3^ Department of Radiation Oncology Duke University School of Medicine Durham NC USA; ^4^ Department of Pathology Duke University Durham NC USA; ^5^ Department of Computational Biology St. Jude Children's Research Hospital Memphis TN USA; ^6^ Department of Molecular Genetics & Microbiology Duke University School of Medicine Durham NC USA

**Keywords:** CDKN1C, HES1, rhabdomyosarcoma, YAP1

## Abstract

Rhabdomyosarcoma (RMS), a cancer characterized by features of skeletal muscle, is the most common soft‐tissue sarcoma of childhood. With 5‐year survival rates among high‐risk groups at < 30%, new therapeutics are desperately needed. Previously, using a myoblast‐based model of fusion‐negative RMS (FN‐RMS), we found that expression of the Hippo pathway effector transcriptional coactivator YAP1 (YAP1) permitted senescence bypass and subsequent transformation to malignant cells, mimicking FN‐RMS. We also found that YAP1 engages in a positive feedback loop with Notch signaling to promote FN‐RMS tumorigenesis. However, we could not identify an immediate downstream impact of this Hippo‐Notch relationship. Here, we identify a HES1‐YAP1‐CDKN1C functional interaction, and show that knockdown of the Notch effector HES1 (Hes family BHLH transcription factor 1) impairs growth of multiple FN‐RMS cell lines, with knockdown resulting in decreased *YAP1* and increased *CDKN1C* expression. *In silico* mining of published proteomic and transcriptomic profiles of human RMS patient‐derived xenografts revealed the same pattern of HES1‐YAP1‐CDKN1C expression. Treatment of FN‐RMS cells *in vitro* with the recently described HES1 small‐molecule inhibitor, JI130, limited FN‐RMS cell growth. Inhibition of HES1 *in vivo* via conditional expression of a *HES1*‐directed shRNA or JI130 dosing impaired FN‐RMS tumor xenograft growth. Lastly, targeted transcriptomic profiling of FN‐RMS xenografts in the context of HES1 suppression identified associations between HES1 and RAS‐MAPK signaling. In summary, these *in vitro* and *in vivo* preclinical studies support the further investigation of HES1 as a therapeutic target in FN‐RMS.

## Graphical abstract

Complementary genetic and pharmacologic approaches were used to demonstrate that inhibition of the transcriptional regulator HES1 increases differentiation and impairs *in vitro* growth in the pediatric cancer rhabdomyosarcoma. Both methods of inhibition were translated to murine xenografts where they effectively limited tumor growth. Created with BioRender.com.

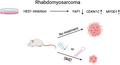

**Abbreviations**
ACTB: actin beta
ANOVA: analysis of variance
CDKN1C: cyclin‐dependent kinase inhibitor 1C
ChIP‐PCR: chromatin immunoprecipitation followed by PCR
CRISPR: clustered regularly interspaced short palindromic repeats
CRISPRi: CRISPR interference method
CSL: alternate name for protein encoded by the RBPJ gene
DLL1/2/4: delta‐like canonical notch ligand 1/2/4
Dox: doxycycline
DUSP4: dual specificity phosphatase 4
FN‐RMS: fusion‐negative rhabdomyosarcoma
FOXO1: forkhead box O1
FP‐RMS: fusion‐positive rhabdomyosarcoma
GSI: gamma secretase inhibitor
H&E: hematoxylin and eosin
HES1: Hes family BHLH transcription factor 1
IC50: half maximal inhibitory concentration
ICD: intracellular domain
IHC: immunohistochemistry
JAG1/2: jagged canonical notch ligand 1/2
JPEG: file extension format created by the Joint Photographic Experts Group
Ki67: proliferation marker based on protein encoded by the MK167 gene
MAML1: mastermind‐like transcriptional coactivator 1
MAPK: mitogen‐activated protein kinase
MHC: myosin heavy chain
MRF4: muscle‐specific regulatory factor 4, encoded by the MYF6 gene
mRNA: messenger RNA
MTT: 3‐(4,5‐dimethylthiazol‐2‐yl)‐2,5‐diphenyltetrazolium bromide
MYOD1: myogenic differentiation 1
MYOG: myogenin
nm
: nanomolar
NRAS: NRAS proto‐oncogene, GTPase
NSG: Nod scid gamma
NT: non‐targeting
P57: alternate name for protein encoded by the CDKN1C gene
PHB2: prohibitin 2
QC: quality control
RAS: proto‐oncogene GTPase family originally named for the rat sarcoma virus
RBPJ: recombination signal binding protein for immunoglobulin kappa J region
RIPA: radioimmunoprecipitation assay buffer
RMS: rhabdomyosarcoma
RNA‐seq: RNA sequencing technique using next‐generation sequencing
ROI: region of interest
RT: room temperature
RT‐qPCR: quantitative reverse transcription PCR
SCID: severe combined immunodeficiency
SD: standard deviation
SEM: standard error of the mean
TAZ: alternate name for protein encoded by the WWTR1 gene
TGF‐beta: transforming growth factor‐beta
Wnt: developmental pathway named for Wingless‐related integration site
WWTR1: WW domain containing transcription regulator 1
YAP1: Yes1 associated transcriptional regulator

## 1 Introduction

Rhabdomyosarcoma (RMS) is the most common soft tissue sarcoma of childhood. Given its expression of myogenic markers, RMS is thought to arise from cell precursors developing aberrantly along the skeletal muscle axis. Originally classified by histopathological subtypes [1], genomic landscape studies now establish molecular stratification that identifies patient subsets as fusion positive (FP) or fusion negative (FN) based on the presence or absence of a *FOXO1* rearrangement [2]. This latter group often has gain‐of‐function mutations in RAS pathway signaling [2, 3, 4]. While FN‐RMS is considered to have a better outcome than FP‐RMS, because of the number of children with FN‐RMS, there are as many deaths [5]. To identify novel therapeutic approaches for FN‐RMS, it is imperative to understand the discrete genetic/epigenetic drivers that can be therapeutically targeted. As is often the case in cancer, particularly those of childhood cancer, FN‐RMS tumorigenesis involves the corruption of multiple developmental pathways [6, 7]. Here, we continue our investigation of the role of dysregulated Notch and Hippo signaling in FN‐RMS tumorigenesis [8].

The Notch signaling pathway is a conserved cell‐to‐cell signaling mechanism that influences cell fate and is crucial to proper myogenesis [9]. Notch signaling begins when a Notch ligand (DLL1,2,4 or JAG1,2) binds to a Notch receptor (NOTCH1‐4), resulting in the release of the Notch intracellular domain (ICD) through a series of proteolytic cleavages. Once free from the membrane‐bound portion of the receptor, ICD translocates to the nucleus where it interacts with transcription factors such as MAML1, CSL, and RBPJ to induce the expression of Notch effectors including transcriptional repressors from the HES/HEY family (reviewed in Ref. [10]). The Hippo pathway is also a conserved developmental signaling network, and influences organ size, tissue regeneration, and tumorigenesis. The core components of the canonical Hippo pathway sequester the transcriptional co‐activators YAP1 and WWTR1 (TAZ) outside the nucleus, limiting their ability to transactivate pro‐growth and anti‐apoptotic signals (reviewed in Ref. [11]). While loss‐of‐function mutations in core Hippo pathway components occur infrequently in cancer [12], YAP1 expression is upregulated in a variety of cancers [13] including in RMS [14, 15, 16].

While identified as separate pathways, Notch and Hippo signaling intersect to control critical myogenic fate decisions including the balance between proliferation and differentiation [17, 18]. YAP1 overexpression in satellite cell (skeletal muscle stem cell [19])‐derived myoblasts blocks myogenic differentiation [18]; YAP1 suppression in FN‐RMS cells induces the transcription of *MYOD1* [20]. HES1 is a direct negative regulator of *MYOD1* transcription, and since HES1 autoregulates its own expression, HES1 and MYOD1 levels oscillate in an inverse fashion [21]. Elevated and prolonged HES1 expression, and consequently prolonged suppression of MYOD1, results in unchecked proliferation. Notch pathway proteins are upregulated in a variety of cancers including FN‐RMS [22]. An examination of 21 primary RMS tumors found *HES1* mRNA increased more than 5‐fold across all samples compared to normal skeletal muscle, and greater than 20‐fold in 13 of 21 samples [23]. *HES1* mRNA levels in the human FN‐RMS cell line, RD, are increased roughly 8‐fold compared to normal skeletal muscle, and inhibition of NOTCH3 signaling in this cell line resulted in reduced growth, increased differentiation, and reduced HES1 expression [24]. Overexpression of HES1 was able to partially mitigate the effects of NOTCH3 inhibition. Very few mutations have been detected in HES1 among pediatric patients [25], suggesting that its dysregulation is at the epigenetic/epigenomic level.

In our prior studies of Hippo signaling in RMS, we found that forced expression of YAP1 in a human myoblast‐based model of FN‐RMS permits bypass of senescence, tolerance of oncogenic RAS, and full transformation to cells able to grow as xenografts mimicking FN‐RMS [20]. In addition, we found that YAP1 engages in a positive feedback loop with Notch signaling, suggesting that Notch effectors impact downstream YAP1, and in FN‐RMS xenografts we found that YAP1 suppression reduced HES1 mRNA [8]. But we did not know the common targets of these interacting pathways. Based on a published YAP1‐driven mouse model of FN‐RMS, in which the induction of YAP1 was associated with loss of *Cdkn1c* expression [15], we hypothesized that a main target of this Notch‐Hippo crosstalk was CDKN1C. Intriguingly, loss‐of‐function of *CDKN1C* is a hallmark of human overgrowth syndromes including Beckwith‐Wiedemann [26] and Costello [27], who are themselves at a higher risk of developing childhood cancer including FN‐RMS [28]. Although rare, heterozygous deletions in *CDKN1C* in non‐syndromic RMS have been reported, suggesting that further study of the impact of loss‐of‐function of this tumor suppressor should be undertaken [29]. Here, we identify a HES1‐YAP1‐CDKN1C functional interaction that is present in human FN‐RMS cell lines and tumor tissue, and show that by blocking HES1 activity via genetic or pharmacologic approaches, we can interfere with FN‐RMS cell growth *in vitro* and tumorigenesis *in vivo*.

## 2 Materials and methods

### 2.1 Generation of cell lines and constructs

HES1 shRNA sequences were generated using the Broad Institute GPP portal (https://portals.broadinstitute.org/gpp/public/). Oligos were obtained from Integrated DNA Technologies (IDT, Coralville, IA, USA) and cloned into pLKO.1 TRC cloning vector (Addgene 10878, Watertown, MA, USA) or Tet‐pLKO‐puro (Addgene 21915). The human RMS cell line RD [30] was a gift from T. Triche (Children's Hospital of Los Angeles, CA, USA) in 2005. SMS‐CTR [31] and Rh36 [32] were gifts from B. Hall (Columbus Children's Hospital, OH, USA) in 2006. Cell line authentication was performed in July 2014 (Rh36) and September 2016 (RD, SMS‐CTR) using STR analysis (Promega GenePrint 10, Promega Corporation, Madison, WI, USA) conducted by the Duke University DNA Analysis Facility (Durham, NC). All cell lines were grown in RPMI‐1640 + 10% FBS in 5% CO_2_.

### 2.2 Quantitative real‐time PCR

RT‐qPCR was performed as described [16]. Measurements were conducted on cells from at least two separate lentivirus HES1shRNA infections. Representative graphs are shown. Primer sets can be found in Table S1.

### 2.3 Pharmacologic agents

JI051 was kindly provided by A. Perron and M. Uesugi (Kyoto University) [33]. JI130 was synthesized by the Duke Small Molecule Synthesis Facility and validated for compound identity and purity (98.9%) by mass spectrometry. Doxycycline hyclate was purchased from Sigma‐Aldrich (D9891, Sigma‐Aldrich, Inc., St. Louis, MO, USA).

### 2.4 MTT assay

RD, SMS‐CTR, and Rh36 cells were seeded in 96‐well plates at 5000, 6000, or 10 000 cells per well, respectively. The following day cells were treated with indicated concentrations of JI051 or JI130. At 48 h, 50 μL of 1 mg·mL^−1^ MTT was added to RD and SMS‐CTR cells and incubated for 3 h. Media was aspirated and 150 μL DMSO was added to each well and mixed 3–4 times. Absorbance was measured at 540 nm (Molecular Devices SpectraMax ABS, Molecular Devices, San Jose, CA, USA). For Rh36 cells, drug‐containing media was replenished and cells were incubated for an additional 48 h before measuring MTT absorbance.

### 2.5 Luciferase assay

The reporter pHES1‐Ub‐luciferase was kindly provided by A. Perron and M. Uesugi. RD cells were seeded in 24‐well format at 5 × 10^4^ cells/well. The experiment utilized JI130, and was otherwise performed as described previously [34]. The assay was performed in duplicate.

### 2.6 Immunoblotting

To generate cell lysates, cell pellets were homogenously suspended on ice by pipette in 50–200 μL RIPA buffer supplemented with protease inhibitor cocktail (ThermoFisher #78430, Thermo Fisher Scientific, Waltham, MA, USA) and 2.5 μL·mL^−1^ benzonase (Millipore #E1014‐5KU, MilliporeSigma, Burlington, MA, USA). Protein concentration was determined in a 96‐well format using the DC protein assay (BioRad #5000111, Hercules, CA, USA) and read with a Molecular Devices SpectraMax ABS instrument. Protein concentrations were normalized and mixed with 4× loading dye (LI‐COR #928‐40004, LI‐COR Biosciences, Lincoln, NE, USA or BioRad #1610747). Thirty to fifty micrograms total protein was loaded per lane on 10% or 4–15% polyacrylamide gel. Proteins were transferred to polyvinylidene difluoride (BioRad Trans‐Blot Turbo #1704150) and membranes blocked with either 5% dry milk or 5% BSA in TBST for 1 h at RT. Primary incubations were performed overnight at 4 °C at the concentrations listed. Secondary incubations were performed at RT for 1 h at 1 : 10 000. Blots were performed in duplicate using cells from two separate lentivirus infections. The following antibodies were used for immunoblotting: anti‐YAP1 (Cell Signaling #4912, 1 : 1000, Cell Signaling Technology, Inc., Danvers, MA, USA), anti‐HES1 (Santa Cruz sc‐166410, 1 : 500, Santa Cruz Biotechnology, Inc., Dallas, TX, USA), anti‐CDKN1C (Cell Signaling #2557, 1 : 1000), anti‐WWTR1/TAZ (Sigma #HPA007415, 1 : 1000, Sigma‐Aldrich, Inc.), anti‐MYOD1 (Dako M3512, 1 : 500, Agilent Dako, Santa Clara, CA, USA), anti‐myogenin (Dako M3559, 1 : 500), and anti‐actin beta (ACTB) (Sigma #A2066, 1 : 1000).

### 2.7 Growth curves

Cells were collected via trypsinization and the resulting suspension was mixed 1 : 1 with trypan blue and counted using a TC10 cell counter (Bio‐Rad). RD and SMS‐CTR cells were seeded in triplicate in 6 cm tissue culture plates at a density of 2.0 × 10^5^ and 2.5 × 10^5^ viable cells per plate respectively. Rh36 cells were seeded in 6‐well plates at 1.14 × 10^5^ viable cells per plate. For each time point, cells were collected and counted as described for seeding. The viable cell count is reported in the growth curve. Only one iteration was done for each cell line as the visual phenotype was striking after multiple separate lentivirus infections.

### 2.8 Apoptosis assays

Cells expressing NT shRNA, HES1sh1, or HES1sh3 were seeded in triplicate in 96‐well format at 10 000 cells per well. NT cells were seeded in two sets of triplicate. The following day staurosporine was added directly to the media of one NT set at an approximate final concentration of 1 μm. Cells were incubated for 1 h at 37 °C after which the plate was allowed to come to RT for 20 min. Media was removed from all conditions and 100 μL of reconstituted Promega Caspase3/7 Assay reagent was added (G8090). The plate was incubated 90 min at RT before being read on a Tecan Spark multimode plate reader (Tecan Group Ltd., Männedorf, Switzerland). The assay was performed in duplicate using cells from two separate lentivirus HES1shRNA infections. Representative graphs are shown.

### 2.9 Differentiation assays

Differentiation assays and MF20 staining were performed as described [35]. The MF20 antibody recognizes all isoforms of myosin heavy chain in differentiated skeletal muscle and was deposited to the Developmental Studies Hybridoma Bank by Fischman, D.A. (DSHB Hybridoma Product MF 20). Positively and negatively stained cells were counted manually with the aid of cell counting software (imagej; NIH, National Institutes of Health, Bethesda, MD, USA) and staining assessment was binary (positive for MF20 staining/not positive for MF20 staining). Eight images were counted per condition.

### 2.10 Xenograft assays

NOD.Cg‐*Prkdc* ^*scid*^ *Il2rg* ^*tm1Wjl*^/SzJ (NSG) and CB17.Cg‐*Prkdc* ^*scid*^ *Lyst* ^*bg‐J*^/Crl (SCID beige) mice were purchased from the Duke Division of Laboratory Animal Resources Animal Breeding Core. Animals were housed in University‐managed facilities according to *The Guide for the Care and Use of Laboratory Animals* and all studies were conducted with the approval of the Duke University IACUC, protocol number A111‐20‐05. Dox‐inducible shRNA xenograft studies utilized 1.25 × 10^6^ RD HES1sh1 or RD HES1sh3 cells/mouse suspended in Matrigel (BD Biosciences, Franklin Lakes, NJ, USA) and implanted subcutaneously into the flanks of immunodeficient NSG mice. Animals were weighed twice weekly and monitored for tumor development. Tumor volume was measured using digital calipers and volume calculated using [(average (length : width))^3^]/2. At approximately 200 mm^3^ average tumor volume, mice were randomly assigned to treatment or control groups and their drinking water was supplemented with 1 mg·mL^−1^ doxycycline (Sigma‐Aldrich) dissolved in 5% w/v sucrose, or 5% w/v sucrose as a control. Mice were sacrificed at a uniform endpoint after 3 weeks treatment before reaching IACUC‐defined maximum tumor burden.

The initial pharmacologic xenograft studies utilized 1.5 × 10^6^ RD cells/mouse suspended in Matrigel (BD Biosciences) and implanted subcutaneously into the flanks of immunodeficient NSG™ mice. Mice were weighed twice weekly and monitored for the emergence of tumors. At approximately 200 mm^3^ average tumor volume, mice were randomly assigned to treatment or vehicle control (DMSO) groups. JI130 dissolved at 50 mg·mL^−1^ in DMSO was administered via intraperitoneal injection at 50 mg·kg^−1^ body weight on days 20–23, 26–27, and 29. Tumor volume was measured as described for the dox‐inducible shRNA study. Mice were sacrificed after 10 days of treatment due to presumed drug‐related toxicity, manifest as weight loss, and tumors were excised and weighed.

The second pharmacologic xenograft study utilized 1.5 × 10^6^ RD cells/mouse suspended in Matrigel (BD Biosciences) and implanted subcutaneously into the flanks of immunodeficient SCID beige mice. At approximately 200 mm^3^ average tumor volume, mice were randomly assigned to treatment or vehicle control (DMSO) groups. JI130 dissolved at 50 mg·mL^−1^ in DMSO was administered via intraperitoneal injection at 50 mg·kg^−1^ body weight on days 21–23, 26, 28, 30, 33, and 35. Mice were sacrificed after 14 days of treatment and tumors were excised and weighed.

### 2.11 Immunohistochemistry (IHC)

Tissue samples were fixed in 10% formalin/70% ethanol and embedded in standard paraffin blocks. Five micrometer sections were mounted and stained with hematoxylin and eosin (H&E) or antibodies. Antibodies and dilutions are listed in Table S2.

### 2.12 Quantification of immunohistochemistry

Ki67‐positive cells from the genetic knockdown tumor xenografts were counted using a VisioPharm workstation. In this digital quantitative image analysis, tumor areas were first identified and outlined in whole slide image (five unique tumors from the genetic knockdown experiment sucrose group and seven unique tumors from the doxycycline group), and APP‐10140 for Ki67 quantification was utilized. The number of cells counted and the percentage of Ki67‐positive cells were collected from the automatic report generated after the APP‐10140 run was completed. An average with SD was reported.

For MYOD1 quantitation, sections from the genetic knockdown tumor xenografts were de‐identified to eliminate scorer bias. Images of the entire tumor section from the second JI130 study were captured using a Leica Aperio GT450 (Leica Biosystems, Deer Park, IL, USA). A representative JPEG file was extracted using aperio imagescope (Leica Biosystems, Deer Park, IL, USA). Using imagej, the JPEG file was split into red, green, and blue channels. The blue channel was used going forward due to its superior contrast. The blue channel was inverted and the background subtracted using a 50‐pixel rolling ball radius. The image was duplicated and the brightness and contrast adjusted using the auto feature. A threshold was set so that most nuclei were selected upon manual inspection. This was applied to make a “binary” image. Next, particles were analyzed using a low cutoff of 30 pixels square to select only nuclei and filter any remaining background. Finally, the designated regions of interest (ROI) were placed over the original inverted blue channel and the intensity within each measured. A weighted average using the area of each measured ROI was generated and reported.

### 2.13 nCounter profiling and analysis

Tumors were homogenized in 1 mL TRIzol reagent (Invitrogen 15596026, Thermo Fisher Scientific) using a Glas‐Col 099D A210101 instrument. After a 5 min RT incubation, 200 μL chloroform was added and the sample was shaken vigorously before a 5 min centrifugation at 4 °C. The aqueous phase was removed and mixed 1 : 1 with 70% ethanol. RNA was isolated using Qiagen RNeasy mini columns (#74106, QIAGEN LLC — USA, Germantown, MD, USA). The QC measures and nCounter experiment on resulting samples were performed by the Duke Sequencing and Genomic Technologies Shared Resource. Pathway analysis and violin plots were generated using the nsolver analysis software (NanoString, Seattle, WA, USA). An nsolver‐independent analysis to identify differentially expressed genes was also carried out. Nanostring nCounter data were processed and normalized using the R package *nanoR* (version 0.1.0, https://github.com/KevinMenden/nanoR). The raw data were parsed using the parseRCC function, followed by background correction and normalization. Downstream differential expression analysis was performed using the R package *limma* (version 3.48.3). The results were robust to different normalization methods applied in *nanoR*.

### 2.14 Statistical analysis

Statistical analysis was performed using graphpad prism (GraphPad, GraphPad Software, San Diego, CA, USA). Unless otherwise noted, data are presented as the mean and SD. One‐way ANOVA and unpaired *T* test were used as appropriate. *P* values were considered significant at *P < 0.05; **P < 0.01; ***P < 0.001; and ****P < 0.0001.

## 3 Results

### 3.1 HES1 suppression impairs FN‐RMS cell growth *in vitro* and reveals a HES1‐YAP1‐CDKN1C functional interaction

To identify candidate downstream genes of YAP1 in FN‐RMS, we interrogated published transcriptome data obtained from tumor‐derived cell lines originating from a conditional genetically engineered mouse model in which *YAP1* could be turned on or off to grow or shrink FN‐RMS tumors [15]. By merging these data with independent datasets containing genes and proteins that are known to impact cellular senescence in mammalian cells [36, 37], we generated a list of 53 candidates (Fig. S1A). Given the role for the cell cycle inhibitor and putative tumor suppressor *CDKN1C* in controlling the switch between myogenic proliferation and differentiation [38], and the mutation of *CDKN1C* in the childhood imprinted disorders Costello syndrome and Beckwith‐Wiedemann syndrome (at risk of developing FN‐RMS [26, 39, 40]), we focused on *CDKN1C* and assessed whether it might be downstream of YAP1 in our system. In a gain‐of‐function approach, using lentiviral transduction we stably ectopically expressed YAP1 (wild‐type or constitutively active mutant YAP1 S127A) in FN‐RMS cells, and found that *CDKN1C* expression was reduced by more than half (Fig. S1B). In a complementary loss‐of‐function approach, we stably expressed two validated shRNAs targeting *YAP1* [20], and found a significant increase in *CDKN1C* (Fig. S1C, left). This connection between YAP1 and CDKN1C in FN‐RMS prompted our investigation of events lying upstream and downstream and their contribution to FN‐RMS tumorigenesis.

**Fig. 1:**
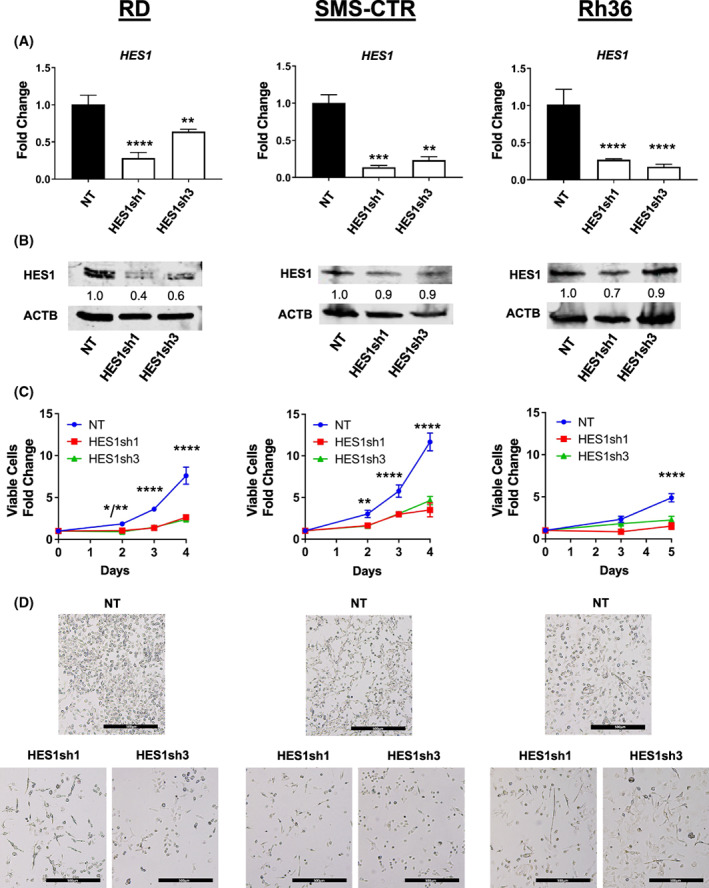
HES1 inhibition impairs FN‐RMS cell growth *in vitro* and alters cellular morphology. (A, B) Two independent lentiviral delivered short hairpin RNAs (shRNAs) were used to knock down Hes family BHLH transcription factor 1 (HES1) in three independent fusion‐negative rhabdomyosarcoma (FN‐RMS) cell lines: RD (left), SMS‐CTR (middle), and Rh36 (right). HES1 knockdown was assessed by quantitative reverse transcription PCR (RT‐qPCR), with *n* = 3; statistical significance determined by one‐way ANOVA with Brown‐Forsythe and Welch tests; graph is representative with error bars indicating standard deviation (SD) of technical triplicate measurements and immunoblotting (*n* = 1) as described in Sections 2 and 3. Densitometry was used to normalize HES1 bands to beta actin (ACTB) loading control. (C) HES1 knockdown impaired RD, SMS‐CTR, and Rh36 cell growth over time as assessed by manual counting (*n* = 3; statistical significance determined by two‐way ANOVA; error bars indicate SD), and (D) led to some cells with elongated morphology reminiscent of myotubes. Images in (D) are 50× total magnification, with scale bars representing 500 μm. *P < 0.05; **P < 0.01; ***P < 0.001, ****P < 0.0001.

To understand what lies upstream of YAP1‐CDKN1C, we again turned to the literature and found that the Notch transcriptional repressor HES1 regulates muscle differentiation in developing mouse embryos through transcriptional control of *Cdkn1c* [38]. Additionally, HES1 is a direct negative transcriptional regulator of *CDKN1C* in hepatocellular carcinoma cells [41]. Using the RD cell line, we tested three independent shRNAs targeting HES1 and assessed efficacy using RT‐qPCR (Fig. S1D) and found that HES1sh1 and sh3 had the strongest effect. Expanding to also include the FN‐RMS cell lines SMS‐CTR and Rh36, we assessed knockdown using HES1sh1 and sh3 and found significant decreases in *HES1* mRNA in all three cell lines, but robust decreases at the protein level only in the RD cell line (Fig. 1A,B; Fig. S2). We also noted that the HES1 band in the RD lysates migrated as a doublet, which may represent HES1 post‐translational modification [42, 43]. The reasons for the technical difficulty in HES1 immunoblotting despite multiple attempts (and in IHC detection, see Section 3.3), and the significance of the HES1 doublet are not known, but future studies would benefit from an epitope tag knocked into the *HES1* genomic locus to more reproducibly follow HES1 at the protein level. Nevertheless, knockdown of HES1 as measured by RT‐qPCR inhibited growth of all three cell lines (Fig. 1C). This was accompanied by morphological changes in the population, manifest as either cellular elongation reminiscent of myotubes (Fig. 1D), or floating cells suggesting cell death. We cannot know (using the methods in this work) whether HES1 depletion caused individual cells to change morphology from round to elongated, or whether only the population of cells without elongated morphology were affected by HES1 depletion, but the latter mechanism would be consistent with an enrichment in elongated cells, and overall decrease in cell number and an increase in apoptotic signal (see ahead to Section 3.2).

**Fig. 2:**
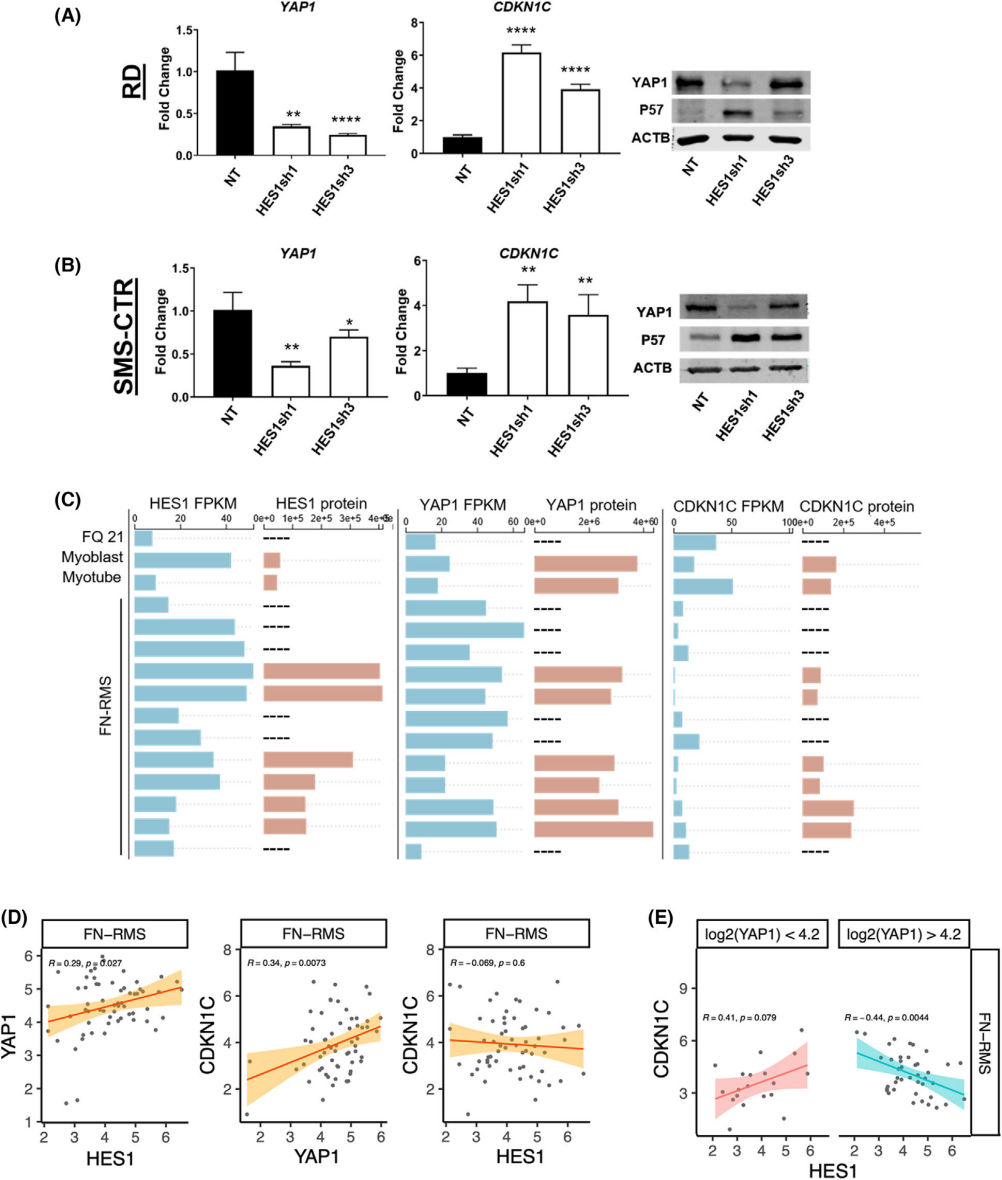
Identification of a HES1‐YAP1‐CDKN1C functional interaction in FN‐RMS. Depletion of Hes family BHLH transcription factor 1 (HES1) results in decreased Yes1 associated transcriptional regulator (*YAP1*) and increased cyclin‐dependent kinase inhibitor 1C (*CDKN1C*) mRNA expression in (A) RD and (B) SMS‐CTR cells, as assessed by quantitative reverse transcription PCR (RT‐qPCR); with *n* = 3; statistical significance determined by one‐way ANOVA with Brown‐Forsythe and Welch tests. Graph is representative with error bars indicating standard deviation (SD) of technical triplicate measurements. Immunoblots (right) (*n* = 2, representative image shown) show the expected increase in P57, the protein encoded by *CDKN1C*, but not the expected decrease in YAP1 protein, potentially reflecting feedback loops controlling YAP1 protein expression (see text). (C) Next‐generation RNA sequencing (RNA‐seq) from 12 fusion‐negative rhabdomyosarcoma (FN‐RMS) human tumor samples shows increased *HES1* (left) and *YAP1* (center) and decreased *CDKN1C* (right) message level (blue). The corresponding protein levels (orange) reflect a similar expression pattern for HES1 protein. Dashes indicate samples in which protein levels were not available. (D) Correlation of gene expression between HES1‐YAP1, YAP1‐CDKN1C, and HES1‐CDKN1C in an independent set of FN‐RMS tumor samples. Analysis was performed using the log2 transformed expression data directly from the NCI Oncogenomics database (https://omics‐oncogenomics.ccr.cancer.gov/). Correlations between HES1‐YAP1 and YAP1‐CDKN1C are positive with a significance level of *P* < 0.05 as determined by correlation tests using Pearson's correlation coefficient. (E) Further analysis of the dataset from (D) using the using the Bioconductor package LiquidAssociation demonstrates a significant three‐way interaction of transcription among the triplet of genes (*P* = 0.006). The three‐way interaction was visualized by dividing the data into low or high expression of YAP1 at a cut‐off of the median expression value of this gene (4.2) and then examining the correlations at different subsets as determined by correlation tests using Pearson's correlation coefficient. In low YAP1, there is a positive correlation between HES1 and CDKN1C; in high YAP1, there is a negative correlation between HES1 and CDKN1C. These results suggest that the relationship between HES1 and CDKN1C might be YAP1‐dependent. *P < 0.05; **P < 0.01; ****P < 0.0001.

To understand what lies downstream of HES1, we found that HES1 suppression resulted in decreased YAP1 (Fig. 2A,B, left; Fig. S3) and increased *CDKN1C* expression at the mRNA level in both RD and SMS‐CTR cells (Fig. 2A,B, middle). The corresponding immunoblots (Fig. 2A,B, right; Fig. S3) showed the predicted increase in P57 protein, but an unexpectedly high YAP1 protein in those cells expressing HES1 sh3 (Fig. 2A,B, right; Fig. S3). We postulate that intracellular feedback loops in Hippo signaling [44], potentially sensitive to the target sequence of the HES1‐directed shRNA and to *YAP1* mRNA levels, resulted in YAP1 protein stabilization. Since WWTR1 is a paralog of YAP1 that has partial overlapping functions, we also examined the impact of HES1 suppression on WWTR1. We found some, but not consistently reduced *WWTR1* mRNA in RD and SMS‐CTR cells without a commensurate decrease in WWTR1 protein (Figs S4A,B and S5), again suggesting compensatory mechanisms. Suppression of WWTR1 using previously validated shRNAs [34] did not increase *CDKN1C* expression (Fig. S1C, right), suggesting that the relationship between CDKN1C and Hippo pathway transcriptional co‐activators may be YAP1‐specific. Finally, to determine whether there might be a relationship between HES1, YAP1, and CDKN1C in human tumor tissue, we examined their expression at the transcript and protein level (when available) in human RMS tumor samples by querying published databases generated for the RMS research community [45]. Across 12 human FN‐RMS samples, HES1 and YAP1 were significantly upregulated compared to fetal muscle tissue, myoblasts, and myotubes, whereas CDKN1C expression was decreased (Fig. 2C). Further, *CDKN1C* transcript levels correlated inversely with *HES1* expression (−0.575, *P* = 0.05). These gain‐ and loss‐of‐function experiments *in vitro* and analysis of available human tumor datasets suggest a functional relationship between HES1, YAP1, and CDKN1C in FN‐RMS, with both HES1 and YAP1 upstream of CDKN1C.

To further probe the relationship between HES1, YAP1, and CDKN1C in FN‐RMS, we interrogated additional human FN‐RMS transcriptomic datasets housed in the NCI Oncogenomics database (https://omics‐oncogenomics.ccr.cancer.gov/), and again found positive correlations between *HES1*‐*YAP1* (Fig. 2D, left) and *YAP1‐CDKN1C* (Fig. 2D, center) at significance levels of *P* < 0.05. The correlation between *HES1‐CDKN1C* was not significantly different from zero in this dataset (Fig. 2D, right). To examine an association between these three genes, we tested whether the relationship between HES1 and CDKN1C might be YAP1‐dependent. We used the Bioconductor package LiquidAssociation to test whether an increase in expression of *YAP1* is associated with a decrease in the correlation between *HES1* and *CDKN1C*. We found a significant three‐way interaction of transcription among the triplet of genes (*P* = 0.006). The three‐way interaction was visualized by dividing the data into low or high expression of *YAP1* at a cut‐off of the median expression value of this gene [log_2_(YAP1) 4.2] and then examining the correlations at different subsets (Fig. 2E). In low *YAP1* (< 4.2), there was a positive correlation between *HES*1 and *CDKN1C*; in high *YAP1*, there was a negative correlation between *HES1* and *CDKN1C*. In summary, these perturbation experiments, analyses of murine and human RMS tumors set, and correlative analyses of a separate, larger data set suggest a functional HES1‐YAP1‐CDKN1C relationship in FN‐RMS that may be sensitive to the level of YAP1 expression.

### 3.2 HES1 suppression induces cell line‐specific myogenic differentiation or apoptosis

**Fig. 3:**
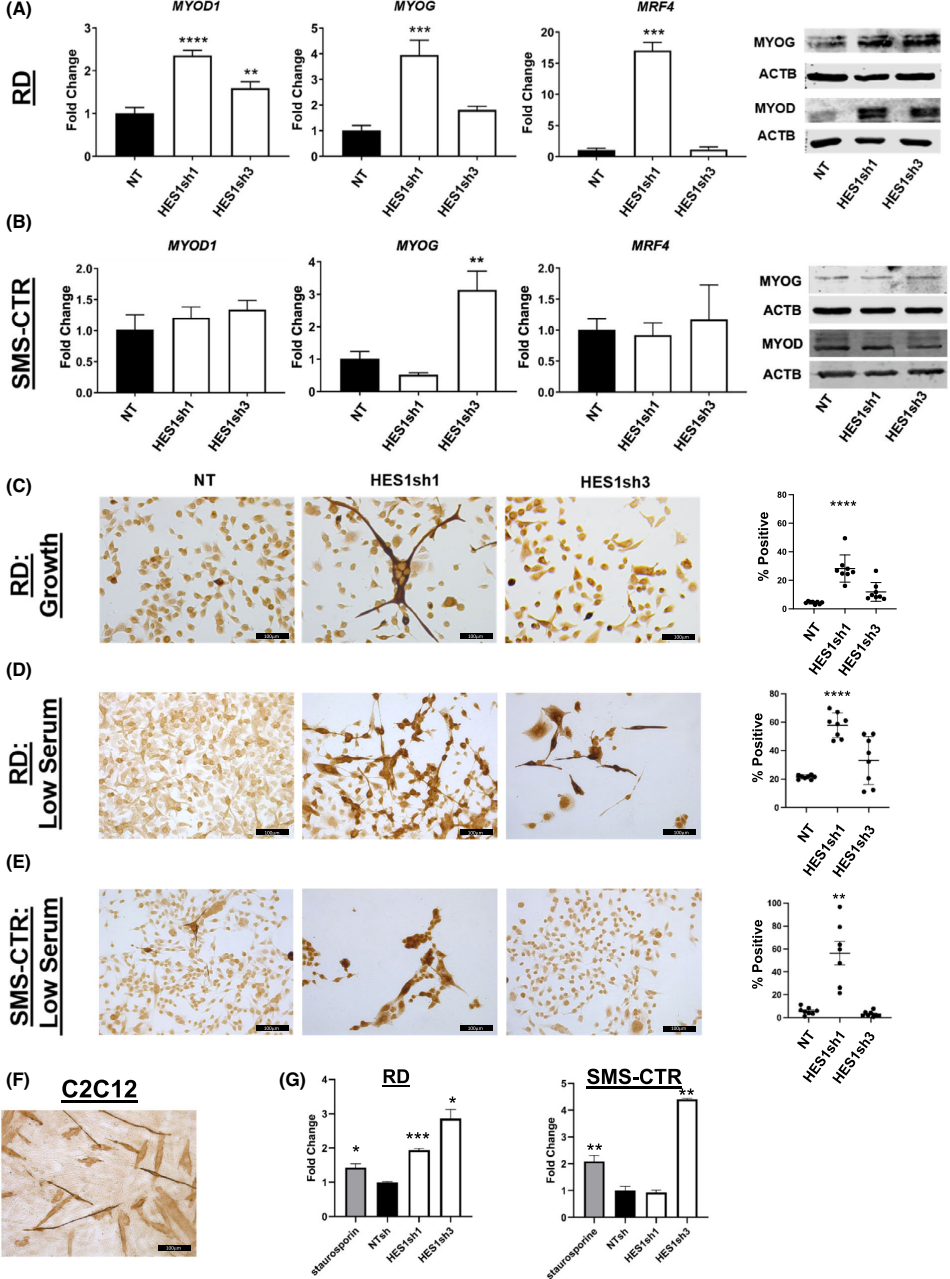
HES1 knockdown *in vitro* increases expression of myogenic markers and can lead to apoptosis. (A) Hes family BHLH transcription factor 1 (HES1) knock down in RD cells increased expression of myogenic differentiation 1 (*MYOD1*), Myogenin (*MYOG*), and muscle‐specific regulatory factor 4 (MRF4, encoded by the *MYF6* gene) at the mRNA level [*n* = 3; statistical significance determined by one‐way ANOVA with Brown‐Forsythe and Welch tests; graph is representative with error bars indicating standard deviation (SD) of technical triplicate measurements], and MYOD1 and MYOG at the protein level (*n* = 2, representative image shown). (B) HES1 knockdown in SMS‐CTR increased expression of MYOG at the mRNA level [*n* = 3; statistical significance determined by one‐way ANOVA with Brown‐Forsythe and Welch tests; graph is representative with error bars indicating standard deviation (SD) of technical triplicate measurements] in cells expressing HES1sh3. This change was not observed at the protein level (*n* = 2, representative image shown). RD cells cultured in (C) standard growth (*n* = 8) or (D) differentiation‐inducing (low serum) conditions (*n* = 8) demonstrate increased myosin heavy chain (MHC) expression in response to HES1 inhibition, with HES1sh1 inducing a stronger phenotype than HES1sh3 that is amplified under differentiation‐inducing (low serum) conditions. (E) SMS‐CTR cells cultured in differentiation conditions (*n* = 8) demonstrate increased MHC positivity in response to HES1sh1. Graphs in C, D, and E depict mean with standard error of the mean (SEM); statistical significance determined by one‐way ANOVA with Brown‐Forsythe and Welch tests. (F) C2C12 murine myoblast cells are included as an MHC positive control (*n* = 3), since they are known to form myotubes under the differentiation conditions used here. Images are 200× total magnification with scale bars representing 100 μm for all images in this figure. (G) The Caspase 3/7 luminescence assay shows strong activity in HES1sh3 expressing cells in both RD and SMS‐CTR (*n* = 2; statistical significance determined by one‐way ANOVA with Brown‐Forsythe and Welch tests; graph is representative with error bars indicating SD of technical triplicate measurements). *P < 0.05; **P < 0.01; ***P < 0.001; ****P < 0.0001.

To better elucidate the phenotypic response of FN‐RMS cells to *HES1* knockdown, we evaluated myogenic and apoptotic phenotypes based on the morphologic changes observed in Fig. 1D. In RD cells, RT‐qPCR and immunoblot of myogenic transcription factors showed a consistent increase in *MYOD1*, *MYOG*, and *MRF4* at the mRNA and protein level (Fig. 3A; Fig. S6), with sh1 showing a more robust response. An increase in MYOD1 expression is consistent with prior ChIP‐PCR studies showing the Hes1 transcriptional repressor binding to the *Myod1* locus in the murine C2C12 myoblast cell line [21]. Even in standard (i.e., not supporting myogenic differentiation) growth media, a significant number of HES1‐knockdown RD cells formed myotube‐like structures and stained positive with an antibody (MF20) against myosin heavy chain (MYH), a marker of terminal myogenic differentiation (Fig. 3C). The MF20 positive stain in RD cells was further enhanced when cells were cultured in myogenic differentiation‐permissive (low serum) conditions for 96 h prior (Fig. 3D). The HES1sh3 RD cells also demonstrated increased MF20 staining under differentiation‐permissive conditions, although not as high as the sh1 cells (Fig. 3D). In contrast, other than a 3‐fold increase in *MYOG* in response to HES1sh3, SMS‐CTR cells did not significantly upregulate myogenic transcription factors (Fig. 3B; Fig. S6), although a low percentage of SMS‐CTR cells expressing HES1shRNA appeared elongated under growth conditions similar to RD cells (Fig. S4C). Culture in differentiation media increased the number of MF20‐positive SMS‐CTR cells in HES1sh1, but not HES1sh3‐expressing cells (Fig. 3E). To investigate our observation of detached, floating cells during cell culture in response to HES1 suppression, we measured caspase 3/7 activity and found that HES1sh1 caused an increased in caspase 3 activity in RD cells, while sh3 caused an increase in both RD and SMS‐CTR cells (Fig. 3G). Throughout these phenotypic studies, we noted variability in amplitude of response to shRNAs, and in correlation between myogenic factor mRNA and protein expression. This variability most likely results from baseline differences in cells (unique genetic backgrounds and mutational profiles responding to genetic perturbation with unique kinetics not captured by our sampling), but we must also consider feedback loops impacting myogenic regulatory factor expression [46] and off‐target effects, a known potential limitation of shRNA technology. In summary, in response to HES1 depletion, RD and SMS‐CTR cells show increases in myogenic differentiation and apoptotic responses, but with some variability between shRNAs.

### 3.3 Genetic inhibition of HES1 impairs FN‐tumorigenesis *in vivo*

**Fig. 4:**
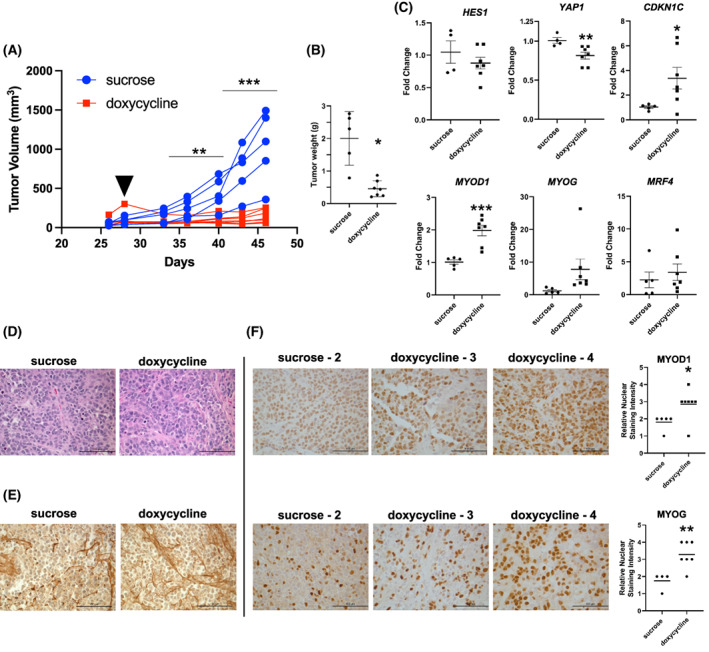
*In vivo* HES1 knockdown impairs tumorigenesis and induces myogenic differentiation of RD cells. (A) RD cell dox‐inducible HES1sh1 xenograft tumor growth over time. See Section 2 for details. The first day of doxycycline treatment is indicated with a solid black triangle. Sucrose control (*n* = 5) and doxycycline groups (*n* = 7) are indicated by blue circles and red squares, respectively. Statistical significance determined by multiple unpaired *t*‐test with Welch's correction. (B) End‐point resected tumor weights for sucrose and doxycycline groups. Statistical significance determined by unpaired *t*‐test with Welch's correction. Horizontal bars indicate mean with standard error of the mean (SEM). (C) Quantitative reverse transcription PCR (RT‐qPCR) of mRNA isolated from tumor xenografts shows a reduction in Hes family BHLH transcription factor 1 (*HES1*) and Yes1 associated transcriptional regulator (*YAP1*), and an increase in cyclin‐dependent kinase inhibitor 1C (*CDKN1C*) and myogenic regulators [myogenic differentiation 1 (*MYOD1*), Myogenin (*MYOG*) and muscle‐specific regulatory factor 4 (encoded by the MYF6 gene *MRF4*)]. Individual points represent average of technical triplicate measurements for control (*n* = 5) and treatment (*n* = 7) groups. Horizontal bars indicate mean with SEM. Statistical significance determined by unpaired *t*‐test with Welch's correction. (D) Hematoxylin and eosin (H&E) staining of sucrose and doxycycline‐treated tumors; sucrose *n* = 5, doxycycline *n* = 7. (E) HES1 immunohistochemistry (IHC) of sucrose and doxycycline‐treated tumors; sucrose *n* = 5, doxycycline *n* = 7. (F) Both MYOD1 (top) and MYOG (bottom) IHC demonstrate increased myogenic protein expression in tumor sections as assessed by quantification on the right side of the panel (see Section 2). Images are 200× total magnification with scale bars representing 100 μm for all images in this figure. Horizontal bars indicate mean with SEM. Statistical significance determined by unpaired *t*‐test with Welch's correction. *P < 0.05; **P < 0.01; ***P < 0.001.

While the identification of a HES1‐YAP1‐CDKN1C functional interaction informed the signaling events altered in FN‐RMS, to translate this to a therapeutic approach, we next investigated the pre‐clinical efficacy of HES1 inhibition *in vivo*. For genetic knockdown, we applied a conditional dox‐inducible system that we developed for FP‐RMS cell lines [16, 34, 47] in which FN‐RMS cells are implanted subcutaneously in immunodeficient mice, and once tumors are palpable, doxycycline (or sucrose control) is added to the drinking water to induce HES1 shRNAs. The HES1sh1‐tet‐on construct was effective *in vitro* at degrading the *HES1* transcripts, inducing the expected downstream changes in *YAP1* and the myogenic markers, and inhibiting cell growth (Fig. S7A,B). Interestingly, this inducible construct did not lead to an increase in *CDK1NC* expression *in vitro* (Fig. S7A), but did lead to an increase in *CDKN1C in vivo* (see ahead to Fig. 4C), suggesting that other forces including but not limited to intensity and duration of knockdown, and potentially tumor microenvironment may impact the efficacy of the dox‐inducible system. We moved forward with this dox‐inducible construct *in vivo*. After almost 3 weeks of dox treatment, the tumors in the sucrose control group had an average volume of > 1000 mm^3^ and an average endpoint weight after necropsy of 2 g, whereas the tumors in the dox treated experimental group had an average volume of < 300 mm^3^ and an endpoint weight after necropsy of 0.45 g (Fig. 4A,B; Fig. S8A). RT‐qPCR analysis of mRNA extracted from the tumors showed *HES1* knockdown with a corresponding decrease in *YAP1* and increase in *CDKN1C*, and myogenic markers *MYOD1*, *MYOG*, and *MRF4* (Fig. 4C). As expected, the tumors histologically resembled FN‐RMS on H&E staining (Fig. 4D). Despite evaluating three different anti‐HES1 antibodies, we could not assay for HES1 protein knockdown via IHC, due to a high background staining (Fig. 4E). To gain insight into the mechanism of decrease in tumor size, we stained the tumor sections for Ki67 and myogenic markers (MYOD1 and MYOG1) as surrogates for proliferation and differentiation. There was no difference in the percent of Ki67‐positive cells between the control and dox groups (76.1 ± 4.5 and 74.1 ± 7.22, respectively). However, the dox group did demonstrate increased expression of MYOD1 and MYOG protein, indicating some degree of myogenic differentiation in response to HES1 genetic suppression (Fig. 4F). In summary, genetic knockdown of HES1 in FN‐RMS tumor xenografts showed inhibition of tumor growth with some induction of myogenic differentiation, supporting the therapeutic potential of targeting the HES1‐YAP1‐CDKN1C functional interaction *in vivo*.

### 3.4 Pharmacologic HES1 inhibitors block FN‐RMS cell growth *in vitro* and *in vivo*

**Fig. 5:**
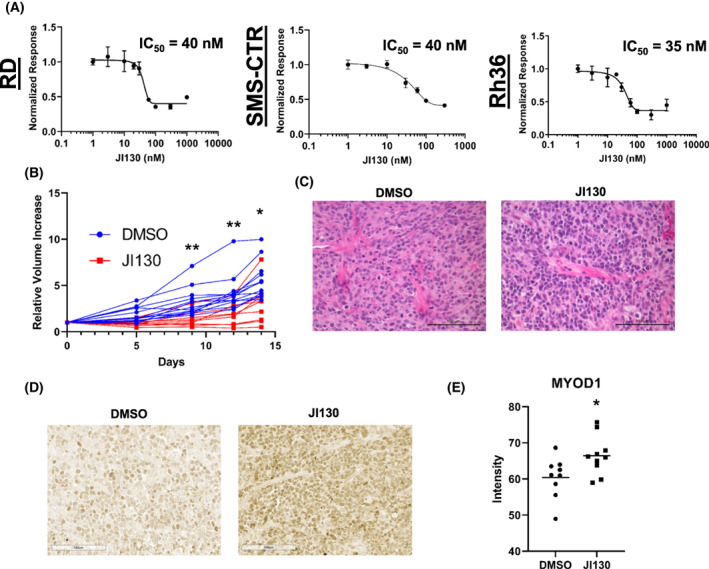
The HES1 pharmacologic inhibitor JI130 suppress FN‐RMS cell growth *in vitro* and *in vivo*. (A) The Hes family BHLH transcription factor 1 (HES1) inhibitor JI130 is effective against RD (left), SMS‐CTR (middle), and Rh36 (right) fusion‐negative rhabdomyosarcoma (FN‐RMS) cells at low nanomolar concentrations *in vitro*. See insets for half‐maximal inhibitory concentration (IC50) values. IC50 determined by non‐linear regression; error bars indicate standard deviation (SD). *n* = 3 (B) JI130 is effective at limiting RD xenograft tumor growth as assessed by measuring calculated tumor volume over time. See Section 2 for details. Statistical significance determined by multiple unpaired *t*‐test with Welch's correction; control *n* = 13, treated *n* = 9. (C) H&E staining of vehicle (DMSO)‐treated and JI130‐treated tumors; control *n* = 9, treated *n* = 10. (D) Immunohistochemistry (IHC) staining for myogenic differentiation 1 (MYOD1) in DMSO‐treated and JI130‐treated tumors; control *n* = 9, treated *n* = 10. (E) imagej quantitation of MYOD1 staining intensity of tumor sections from (D). Horizontal bars indicate mean with standard error of the mean (SEM). Statistical significance determined by unpaired *t*‐test with Welch's correction; control *n* = 9, treated *n* = 10. Images are 200× total magnification with scale bars representing 100 μm for all images in this figure. *P < 0.05; **P < 0.01.

To take advantage of the translational opportunities presented by this HES1‐YAP1‐CDKN1C relationship, we sought to pharmacologically block the most upstream component, HES1. In 2018, Perron et al. performed a small molecule screen identifying compound JI051 and its derivative JI130 that inhibited HES1 activity via an unexpected mechanism. These compounds stabilized the interaction of HES1 with the PHB2 chaperone [33], preventing HES1 from entering the nucleus and impairing its ability to repress transcription. Evaluation of JI051 in our cell system showed that it blocked HES1 transcriptional repression as assessed by a luciferase reporter assay in RD cells (Fig. S9A,B), but also inhibited growth of RD, SMS‐CTR, and Rh36 cells (Fig. S9C,D), with IC50s in the 30–50 nm range. In preparation for *in vivo* studies, we examined the JI051 derivative, JI130, which Perron et al. [33] found in pancreatic cell lines to be 6‐fold more potent than JI051. Again, we found IC50s in the low‐nanomolar range (Fig. 5A). Using our xenograft system, we tested the effect of JI130 *in vivo* by randomizing RD tumor xenograft‐bearing mice to DMSO vehicle or JI130 using the established dosing regimen [33]. Unlike Perron et al., we found some toxicity, as our cohort experienced weight loss (Fig. S8B), and we therefore truncated treatment to 10 days. Similar to the genetic inhibition studies, HES1 pharmacologic inhibition by JI130 inhibited tumor xenograft growth over time (Fig. S8C,D), with lower average tumor weight after necropsy (Fig. S8E). We attempted histochemical and molecular analysis of the tumors from this experiment, but the tumor sections were degraded and unsuitable for analysis. We repeated the experiment with a similar design, except using *scid*‐beige in lieu of NSG™ and dosing three times weekly, and again found inhibition of tumor xenograft growth (Fig. 5B), with H&E (Fig. 5C) showing expected RMS cells but no obvious morphologic difference between vehicle and treatment groups. IHC revealed a modest but statistically significant increase in MYOD1 staining in the JI130 group (Fig. 5D,E), again suggesting myogenic differentiation. These genetic and pharmacologic studies together suggest that blockade of HES1 in FN‐RMS could be a viable therapeutic approach.

### 3.5 mRNA profiling to investigate signaling changes downstream of HES1 knockdown

To further investigate the mechanism of tumor growth inhibition *in vivo*, we performed mRNA profiling of the knockdown xenograft tumors using the Nanostring nCounter^®^ platform and Tumor Signaling 360™ panel (Table S3). In agreement with our RT‐qPCR data, nCounter analysis identified *MYOD1* and *MYOG* as two of the most significantly differentially expressed genes in response to HES1 suppression (Fig. S10). Similarly, the Notch ligand DLL1 was significantly expressed in the treated group. This is consistent with the recent finding that HES1 and MYOD1 can bind to an intronic enhancer region of *DLL1*, with HES1 inhibiting but MYOD1 promoting *DLL1* expression. Coordinated oscillation of these three genes is critical for equilibrium between stemness and differentiation in myogenic progenitors and adult muscle stem cells [48]. Upregulation of JAG1 in this dataset is also intriguing, since Jag1 was recently found to modulate the oscillatory Dll1‐Hes1 circuit in murine pancreatic progenitors [49]. The nCounter analysis also showed changes in expression in several RAS‐MAPK pathway genes, including downregulation of *NRAS* (which in RD cells contains an oncogenic Q61H mutation [50]) and upregulation of the MAPK regulator dual specificity phosphatase 4 (*DUSP4*) [51]. Hierarchical clustering as part of a pathway analysis (Fig. S11) identified two subgroups within the doxycycline‐treated tumors (Fig. S12A,B). While the small sample number created by splitting of the treatment group precludes statistically significant comparisons between the two groups, it is compelling that the tumors with the greatest increases in *CDKN1C* and *MYOG* as measured by RT‐qPCR (Fig. S12C,D), suggesting they are the most differentiated, also had the smallest endpoint tumor volume (Fig. S12E). The modest decrease in *HES1* tumor mRNA but robust increase in *CDKN1C* as measured by the nCounter experiment (Fig. S12F,G) likely reflects tumor cell heterogeneity, i.e., some RMS cells become resistant to the shRNA and continue dividing as the xenograft experiment progresses as we have seen previously [47], while others cease dividing and express *CDKN1C*. In summary, these unbiased analyses of HES1‐suppressed tumors reinforce the role of a HES1‐YAP1‐CDKN1C functional interaction in FN‐RMS, but also point to the future investigation of RAS‐MAPK signaling in this interaction.

## 4 Discussion

It has become clear that dysregulation of developmental pathways contributes considerably to oncogenic signaling in FN‐RMS. This includes Notch, Hippo, Wnt, Hedgehog and TGF‐beta and the dysregulation of these developmental pathways in FN‐RMS has been reviewed previously [7, 52]. In addition to specific findings of dysregulated Notch and Hippo in FN‐RMS, prior studies also identified a role for crosstalk between Notch and Hippo in FN‐RMS stemness, using FN‐RMS spheroid cultures *in vitro* [8]. Regarding the link between Notch or Hippo signaling and CDKN1C, prior studies in non‐transformed human mammary cells [53] and murine lymphangiogenesis models [54] identified a role for YAP1 in downregulating CDKN1C, and in human hepatocellular carcinoma primary tumors and cell lines demonstrated a strong inverse relationship between HES1 protein and *CDKN1C* mRNA levels, with HES1 loss‐of‐function or P57 (the protein name for *CDKN1C* gene) gain‐of‐function inducing a senescence phenotype [41]. The current work thus places Notch‐Hippo crosstalk in FN‐RMS in a biologically relevant framework, where Notch‐Hippo signaling converges on CDKN1C to make cellular decisions about continuing through the cell cycle versus initiating a myogenic differentiation program. This relationship is likely conserved in other cancers but must be examined on a case‐by‐case basis.

Targeting developmental pathways in human cancer is not a new approach, and most attempts to inhibit Notch signaling have utilized γ‐secretase inhibitors (GSIs). However, γ‐secretase has over 90 protein targets in addition to the Notch receptors [55], leading to a high level of toxicity, particularly in the gut, for first‐generation GSIs [56]. Here we take advantage of work done by Perron et al. in pancreatic cancer models to show that the small molecule HES1 inhibitor JI130 inhibits growth of human FN‐RMS tumor cells and xenografts. We postulated that targeting the Notch pathway further downstream than the Notch receptor level could lead to greater selectivity and less off‐target effects, and JI130 offers proof of concept that this is possible and that this chemical space is worth further exploration. Alternative approaches toward inhibiting HES1 protein function include small molecules inhibiting its dimerization [57] and transcription [58]. While we observed weight loss in our mouse studies, Perron et al. did not, suggesting that this side effect could be related to mouse strain (NSG versus nude mice) and/or related to differences in human cell lines studied. The fact that the genetic depletion of HES1 had a greater impact on tumor size compared to JI130 might also mean that this particular agent will not be able to fully inhibit HES1 to the extent needed due to toxicity. Toxicity observed in early studies of developmental pathway inhibitors can be overcome, as seen by the ongoing trials of a next‐generation GSI nirogacestat in desmoid tumors in children and adolescents (ClinicalTrials.gov Identifier NCT04195399). However, because children and adolescents are still growing, and the impact of modulating these pathways can have serious effects on linear growth and endocrine function [59], these agents must be evaluated with care.

Our study raises several questions that will require further investigation. For example, why do different FN‐RMS cell lines manifest different growth inhibitory phenotypes even when the same oncogenic signal (i.e., HES1) is blocked in the same way? We speculate that it may be related to unique genetic background and/or mutational profile. For example, RD cells harbor an *NRAS* mutation while SMS‐CTR has an *HRAS* mutation, and different RMS cell lines have demonstrated differential expression of Notch pathway components [24]. Indeed, we have found differences between HEY1 and HES1 behavior in FN‐RMS. In prior experiments [8], we found that YAP1 suppression in RD and SMS‐CTR rhabdospheres *in vitro* caused a decline in *HEY1*, but not *HES1*. However, *in vivo* in SMS‐CTR tumor xenografts, YAP1 suppression was associated with a decline in *HES1*. Thus, even HES1 and HEY1 do not always track together and need to be studied independently, and their expression and function may differ depending upon cell line and tumor microenvironment. There is also the 40–50% of FN‐RMS cases that have no RAS or RAS‐pathway mutations; future single‐cell RNA‐seq studies could illuminate the impact of genetic background on Notch pathway inhibition. Other possible sources of variability in the response to HES1 depletion include the kinetics of response to shRNA suppression in different FN‐RMS cell lines, and the known limitation of off‐target effects from shRNAs. Thus it will be important to study additional FN‐RMS cell lines including patient‐derived xenograft systems, and employ alternate loss‐of‐function approaches to deplete HES1 including CRISPR or CRISPRi. It will also be important to investigate the relationship between HES1, YAP1, and CDKN1C in FP‐RMS. The FP‐RMS cell lines RHJT and Rh28 exhibit a 10‐fold and 5‐fold increase in HES1 mRNA compared to skeletal muscle, respectively [23], and RHJT FP‐RMS cells cultured under low serum conditions for 72 h showed an increase in MHC expression when forced to express a dominant negative HES1 construct, suggesting that HES1 is necessary to protect against myogenic differentiation. However, not all cell systems are sensitive to HES1 depletion. For example, in studies of lymphocyte development and transformation, HES1 was dispensable for Notch‐dependent thymocyte maturation [60] and some T‐cell responses [61], so the role of HES1 in each biological system must be examined independently.

Last, and the direction of our future work, we query the molecular nature of YAP1 and HES1 convergence on *CDKN1C*. While HES1 is known to bind directly to the *CDKN1C* promoter [41, 62], we show here in RD cells that YAP1 overexpression inhibits *CDKN1C* expression without affecting HES1 expression (Fig. S13). Does YAP1, traditionally thought of as a co‐activator, partner with HES1 to repress *CDKN1C* transcription or more likely, does it activate TLE1 or a different protein partner of HES1? The *in silico* analyses finding a three‐way interaction between HES1, YAP1, and CDKN1C suggest that the relationship between *HES1* and *CDKN1C* might depend upon *YAP1* expression levels. The relationship of this axis to MYOD1 expression and activity is also important to understand. For example, P57 protein interacts physically with MYOD1, resulting in the stabilization of both proteins [63]. Although this protein–protein interaction could be important after induction of *CDKN1C*, the fact that HES1 and MYOD1 protein oscillation regulates the maintenance of activated muscle stem cells [21], and that YAP1 protein expression also oscillates [64] suggests a more complex impact of HES1 and YAP1 expression on both MYOD1 and CDKN1C. Future studies examining the timing of protein‐protein and protein‐chromatin interaction in specific genomic loci will be required to understand the precise roles of HES1, YAP1, and CDKN1C in both FN‐RMS and FP‐RMS, in additional cell lines and primary patient tissue, and the impact on cell fate decisions governing cell proliferation, survival, and stemness versus induction of myogenic differentiation. Since our nCounter data reveal significant perturbation to transcript levels of RAS‐MAPK pathway components, this suggests a critical role of HES1 in their regulation. Future investigations will mechanistically interrogate the transcriptional program governed by HES1 and its pivotal role in FN‐RMS tumorigenesis.

## 5 Conclusions

Fusion‐negative rhabdomyosarcoma (FN‐RMS) is a childhood cancer of skeletal muscle histogenesis that requires further investigation to identify its molecular underpinnings and discover new therapeutic targets. Here, we identify a HES1‐YAP1‐CDKN1C functional interaction that supports FN‐RMS tumor cell growth and tumorigenesis. Complementary genetic and pharmacologic approaches to block HES1 increased myogenic differentiation and impaired *in vitro* cell and *in vivo* tumor growth. Unbiased mRNA profiling revealed a relationship between HES1 and the RAS‐MAPK pathway. Future investigations will interrogate the molecular nature of YAP1 and HES1 convergence on CDKN1C, the transcriptional program governed by HES1 in FN‐RMS tumorigenesis, and the impact on FN‐RMS cell fate decisions, informing potential therapeutic opportunities for this childhood cancer.

## Conflict of interest

DGK is a cofounder of and stockholder in XRAD Therapeutics, which is developing radiosensitizers. DGK is a member of the scientific advisory board for and owns stock in Lumicell Inc, a company commercializing intraoperative imaging technology. None of these affiliations represents a conflict of interest with respect to the work described in this manuscript. DGK is a coinventor on a patent for a handheld imaging device and is a coinventor on a patent for radiosensitizers. XRAD Therapeutics, Merck, Bristol Myers Squibb, and Varian Medical Systems provide research support to DGK, but this did not support the research described in this manuscript.

## Author contributions

ARK and CML conceived of and acquired funding for the project. ARK, KMO, and RCB developed methodology. ARK, KMO, CC, and XC performed the investigations. ARK, CC, XC, P‐HC, and J‐TAC performed formal analysis. CC and XC curated data and provided data visualization. CML administered and supervised the project. CML and DGK provided resources. ARK and CML wrote the original draft. All authors reviewed and edited the document. Author contributions are listed according to CRediT taxonomy (https://credit.niso.org/).

### Peer review

The peer review history for this article is available at https://publons.com/publon/10.1002/1878‐0261.13304.

## Supporting information


**Fig. S1.** YAP1 promotes CDKN1C downregulation, and assessment of HES1 shRNA constructs.
**Fig. S2.** Full immunoblots corresponding to Figure 1.
**Fig. S3.** Full immunoblots corresponding to Figure 2A and Figure 2B.
**Fig. S4.** HES1 knockdown inconsistently suppresses WWTR1 expression. HES1 suppression does not significantly induce differentiation of SMS‐CTR cells under normal growth conditions.
**Fig. S5.** Full immunoblots corresponding to Supplemental Figure S4.
**Fig. S6.** Full immunoblots corresponding to Figure 3A,B.
**Fig. S7.** *In vitro* validation of doxycycline‐inducible HES1shRNA.
**Fig. S8.** Tumor xenograft resections and changes in mouse weight during *in vivo* genetic and pharmacologic HES1 inhibition.
**Fig. S9.** Effect of the HES1 pharmacologic inhibitor J1051 in HES1 luciferase reporter assays and cell viability *in vitro*.
**Fig. S10.** nCounter volcano plot.
**Fig. S11.** nCounter mRNA pathway profiling.
**Fig. S12.** nCounter mRNA pathway analysis hierarchically clusters the doxycycline treatment group as more or less differentiated.
**Fig. S13.** *YAP1* overexpression reduces *CDKN1C* but not *HES1* transcript levels.

**Table S1.** RT‐qPCR primers.

**Table S2.** IHC antibodies.

**Table S3.** Full list of mRNA profiling of the HES1 knockdown RD xenograft tumors using the Nanostring nCounter^®^ platform and Tumor Signaling 360™ panel.

## Data Availability

Additional data is available as Supplementary Information.
