# Supplementary material for: Identification and targeting of a HES1‐YAP1‐CDKN1C functional interaction in fusion‐negative rhabdomyosarcoma
Source: Mol Oncol. 2022 Aug 29;16(20):3587–605. doi: 10.1002/1878-0261.13304 (PMC9580881; doi:10.1002/1878-0261.13304)
Supplement: Supplementary file 1 — Fig. S1. YAP1 promotes CDKN1C downregulation, and assessment of HES1 shRNA constructs. Fig. S2. Full immunoblots corresponding to Figure 1. Fig. S3. Full immunoblots corresponding to Figure 2A and Figure 2B. Fig. S4. HES1 knockdown inconsistently suppresses WWTR1 expression. HES1 suppression does not significantly induce differentiation of SMS‐CTR cells under normal growth conditions. Fig. S5. Full immunoblots corresponding to Supplemental Figure S4. Fig. S6. Full immunoblots corresponding to Figure 3A,B. Fig. S7. In vitro validation of doxycycline‐inducible HES1shRNA. Fig. S8. Tumor xenograft resections and changes in mouse weight during in vivo genetic and pharmacologic HES1 inhibition. Fig. S9. Effect of the HES1 pharmacologic inhibitor J1051 in HES1 luciferase reporter assays and cell viability in vitro. Fig. S10. nCounter volcano plot. Fig. S11. nCounter mRNA pathway profiling. Fig. S12. nCounter mRNA pathway analysis hierarchically clusters the doxycycline treatment group as more or less differentiated. Fig. S13. YAP1 overexpression reduces CDKN1C but not HES1 transcript levels. [file MOL2-16-3587-s004.pptx]

## Slide 1
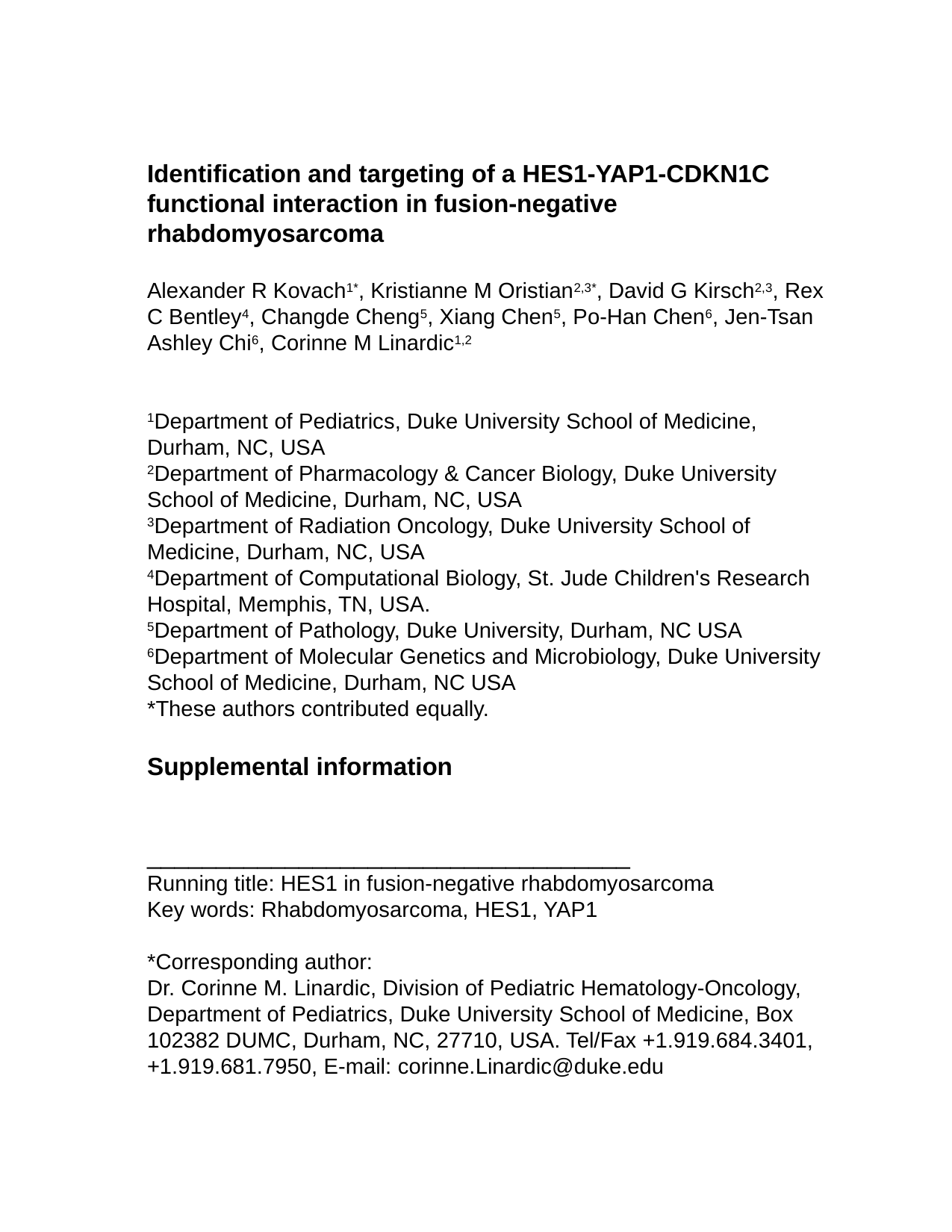

Identification and targeting of a HES1-YAP1-CDKN1C functional interaction in fusion-negative rhabdomyosarcoma
Alexander R Kovach1*, Kristianne M Oristian2,3*, David G Kirsch2,3, Rex C Bentley4, Changde Cheng5, Xiang Chen5, Po-Han Chen6, Jen-Tsan Ashley Chi6, Corinne M Linardic1,2
1Department of Pediatrics, Duke University School of Medicine, Durham, NC, USA
2Department of Pharmacology & Cancer Biology, Duke University School of Medicine, Durham, NC, USA
3Department of Radiation Oncology, Duke University School of Medicine, Durham, NC, USA
4Department of Computational Biology, St. Jude Children's Research Hospital, Memphis, TN, USA.
5Department of Pathology, Duke University, Durham, NC USA
6Department of Molecular Genetics and Microbiology, Duke University School of Medicine, Durham, NC USA
*These authors contributed equally.
Supplemental information
___________________________________
Running title: HES1 in fusion-negative rhabdomyosarcoma
Key words: Rhabdomyosarcoma, HES1, YAP1
*Corresponding author:
Dr. Corinne M. Linardic, Division of Pediatric Hematology-Oncology, Department of Pediatrics, Duke University School of Medicine, Box 102382 DUMC, Durham, NC, 27710, USA. Tel/Fax +1.919.684.3401, +1.919.681.7950, E-mail: corinne.Linardic@duke.edu

## Slide 2
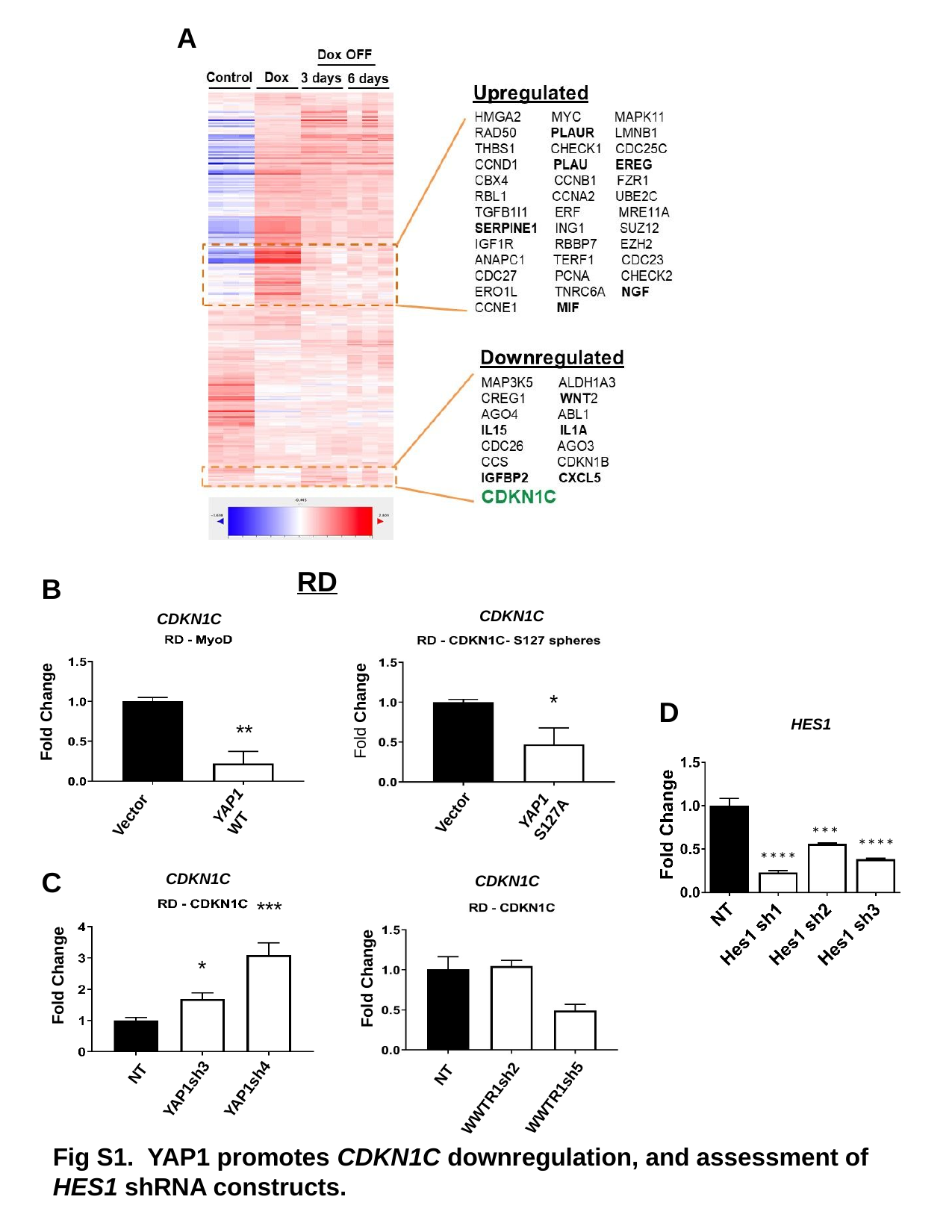

A
RD
B
CDKN1C
*
Fold Change
YAP1 S127A
Vector
CDKN1C
Fold Change
**
YAP1
WT
Vector
D
HES1
***
****
****
C
CDKN1C
***
*
Fold Change
NT
YAP1sh4
YAP1sh3
CDKN1C
Fold Change
NT
WWTR1sh5
WWTR1sh2
Fig S1.  YAP1 promotes CDKN1C downregulation, and assessment of HES1 shRNA constructs.

## Slide 3
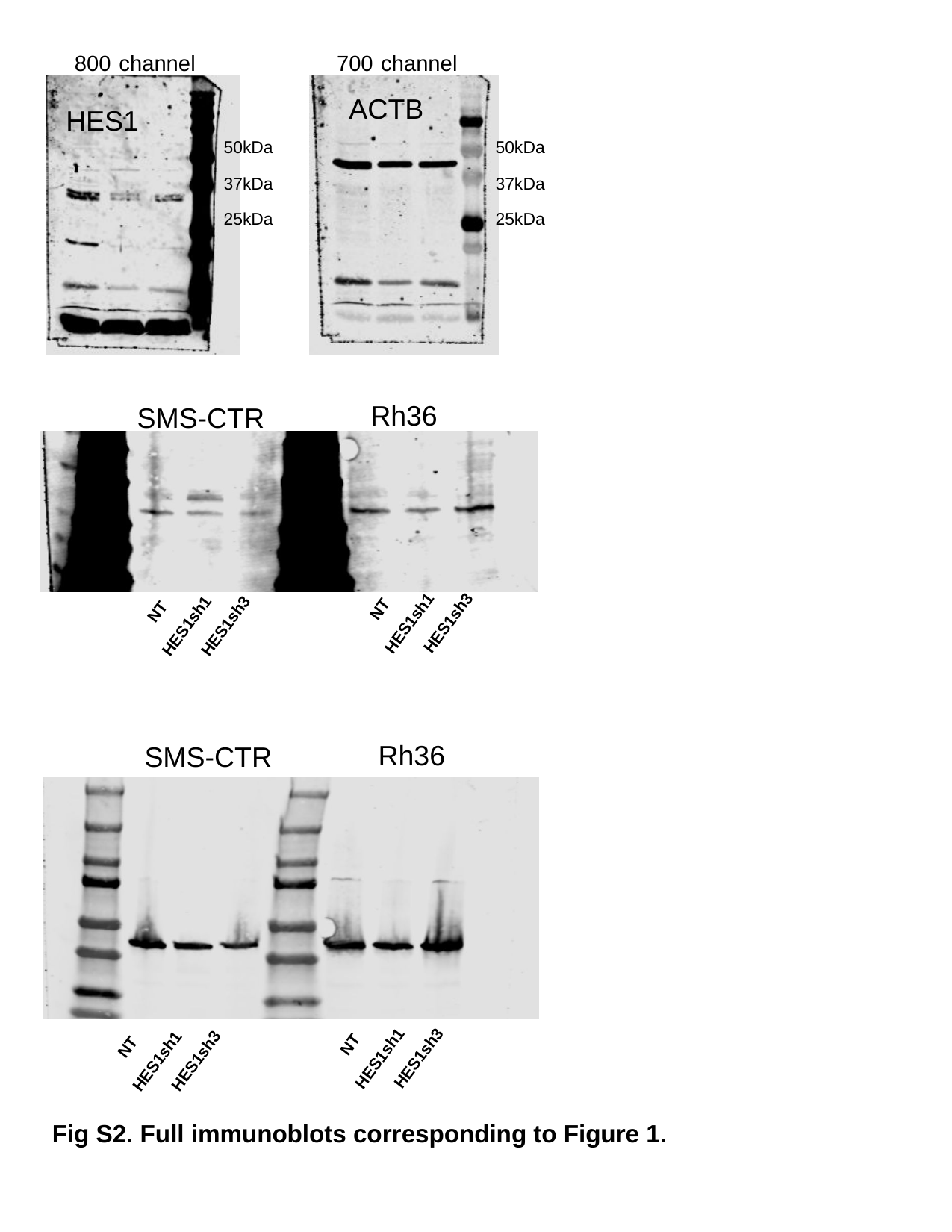

800 channel
HES1
50kDa
37kDa
25kDa
700 channel
ACTB
50kDa
37kDa
25kDa
Rh36
SMS-CTR
NT
NT
HES1sh3
HES1sh1
HES1sh3
HES1sh1
Rh36
SMS-CTR
NT
NT
HES1sh3
HES1sh1
HES1sh3
HES1sh1
Fig S2. Full immunoblots corresponding to Figure 1.

## Slide 4
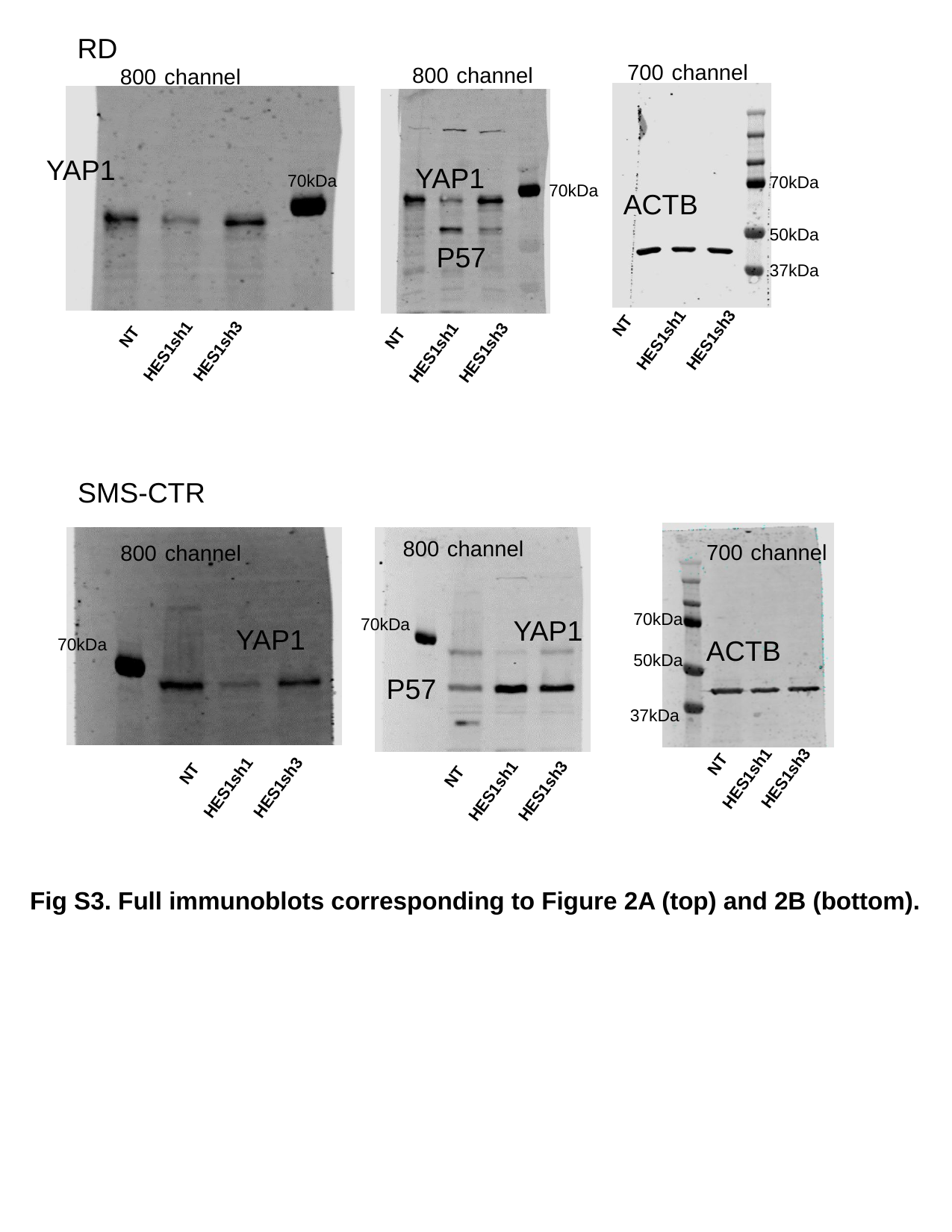

RD
700 channel
800 channel
800 channel
70kDa
ACTB
50kDa
37kDa
NT
HES1sh3
HES1sh1
YAP1
YAP1
70kDa
70kDa
P57
NT
NT
HES1sh3
HES1sh1
HES1sh3
HES1sh1
SMS-CTR
800 channel
700 channel
70kDa
ACTB
50kDa
37kDa
NT
HES1sh3
HES1sh1
800 channel
70kDa
YAP1
YAP1
70kDa
P57
NT
NT
HES1sh3
HES1sh1
HES1sh3
HES1sh1
Fig S3. Full immunoblots corresponding to Figure 2A (top) and 2B (bottom).

## Slide 5
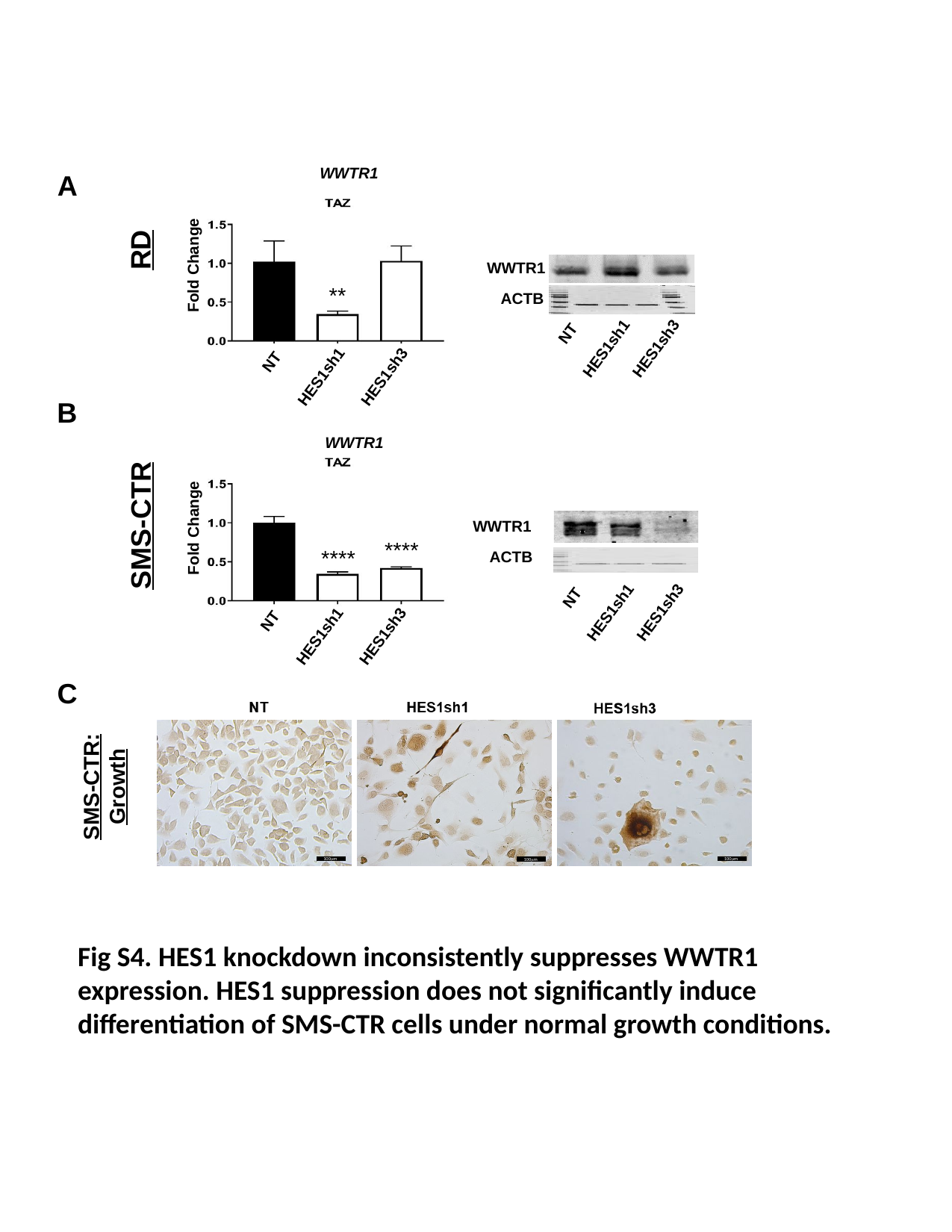

WWTR1
Fold Change
**
NT
HES1sh3
HES1sh1
A
RD
WWTR1
ACTB
NT
HES1sh3
HES1sh1
B
WWTR1
Fold Change
NT
HES1sh3
HES1sh1
****
****
SMS-CTR
WWTR1
ACTB
NT
HES1sh3
HES1sh1
C
SMS-CTR:
Growth
100μm
100μm
100μm
Fig S4. HES1 knockdown inconsistently suppresses WWTR1 expression. HES1 suppression does not significantly induce differentiation of SMS-CTR cells under normal growth conditions.

## Slide 6
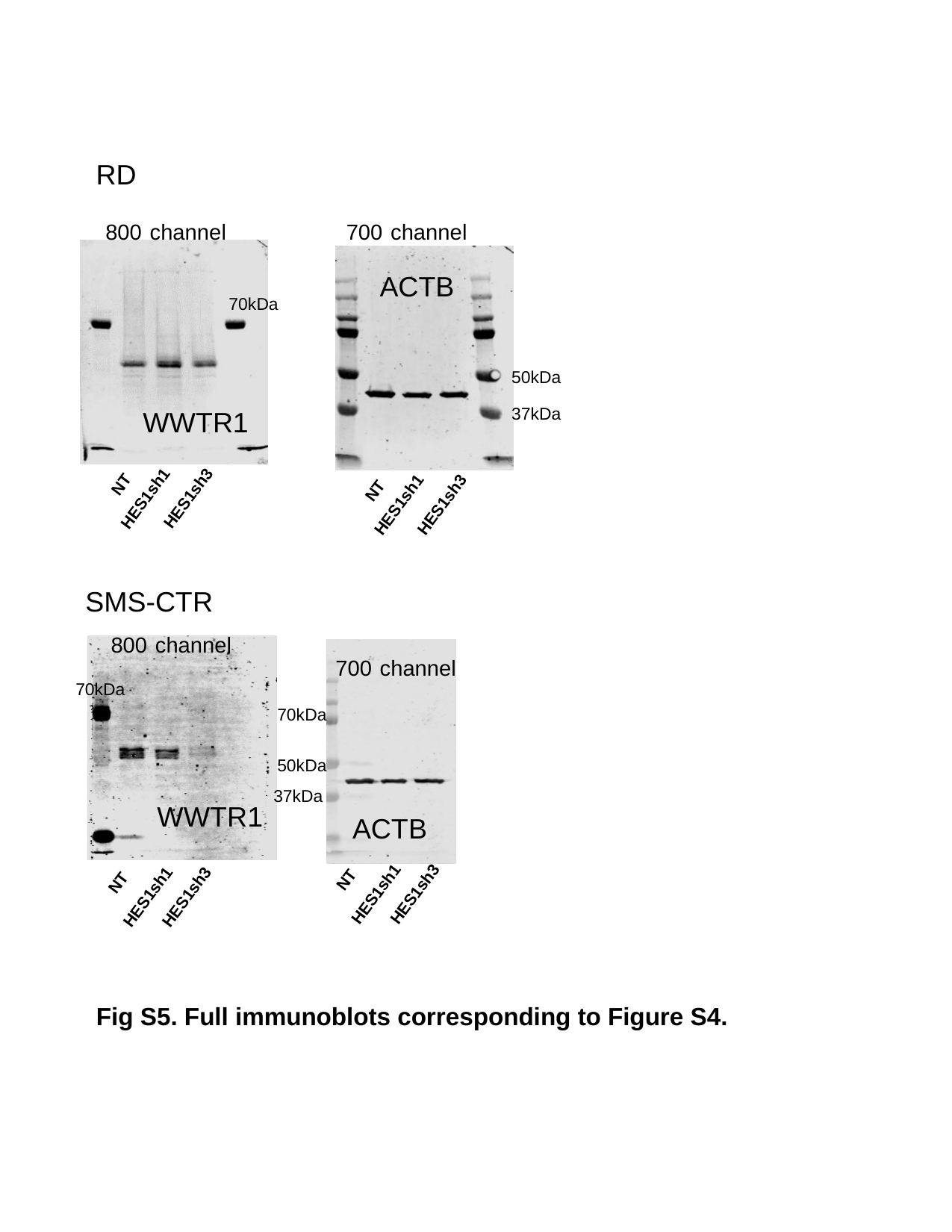

RD
800 channel
70kDa
WWTR1
NT
HES1sh3
HES1sh1
700 channel
ACTB
50kDa
37kDa
NT
HES1sh3
HES1sh1
SMS-CTR
800 channel
700 channel
70kDa
70kDa
50kDa
37kDa
WWTR1
ACTB
NT
NT
HES1sh3
HES1sh1
HES1sh3
HES1sh1
Fig S5. Full immunoblots corresponding to Figure S4.

## Slide 7
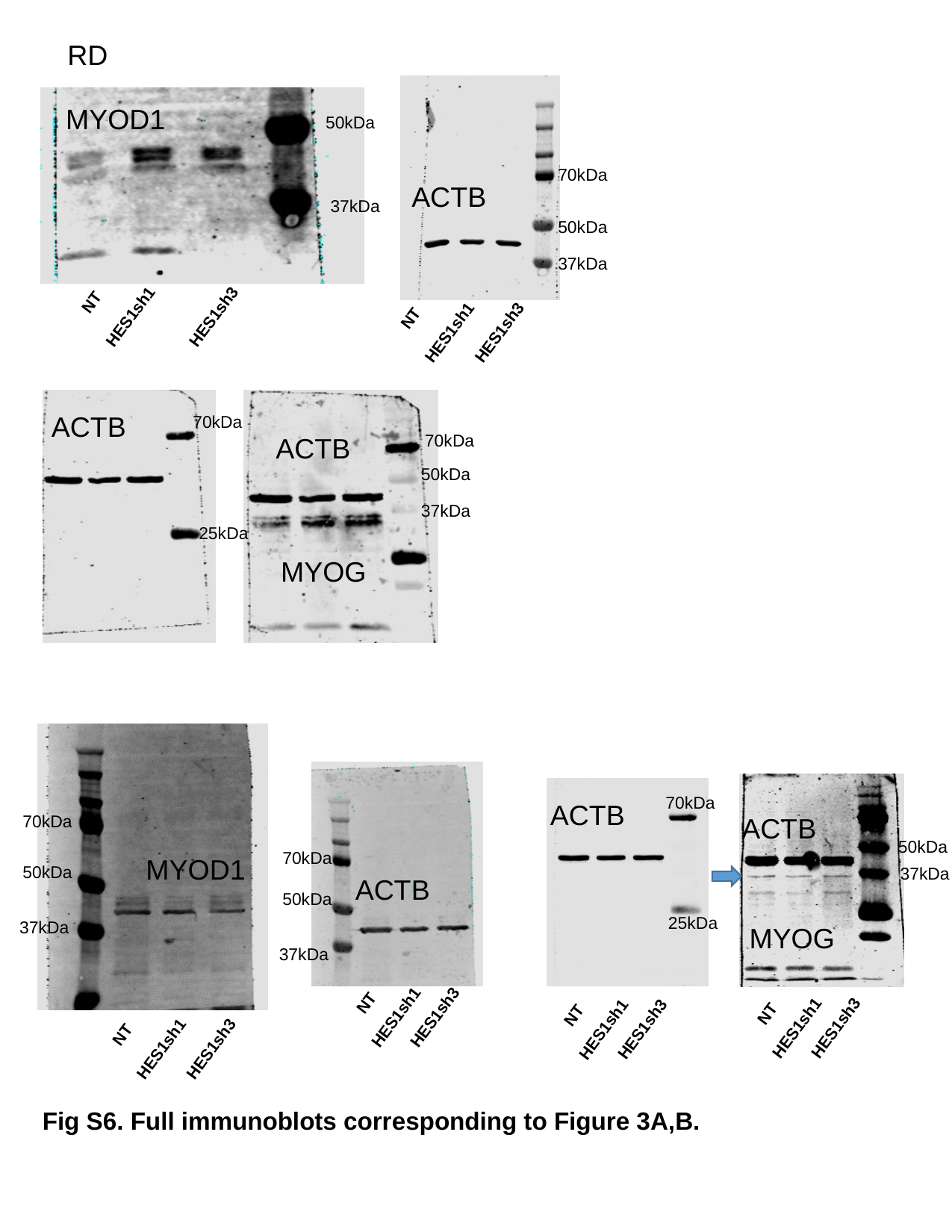

RD
70kDa
ACTB
50kDa
37kDa
NT
HES1sh3
HES1sh1
MYOD1
50kDa
37kDa
NT
HES1sh3
HES1sh1
ACTB
70kDa
25kDa
70kDa
ACTB
50kDa
37kDa
MYOG
70kDa
MYOD1
50kDa
37kDa
NT
HES1sh3
HES1sh1
70kDa
ACTB
50kDa
37kDa
NT
HES1sh3
HES1sh1
NT
HES1sh3
HES1sh1
NT
HES1sh3
HES1sh1
70kDa
ACTB
ACTB
50kDa
37kDa
25kDa
MYOG
Fig S6. Full immunoblots corresponding to Figure 3A,B.

## Slide 8
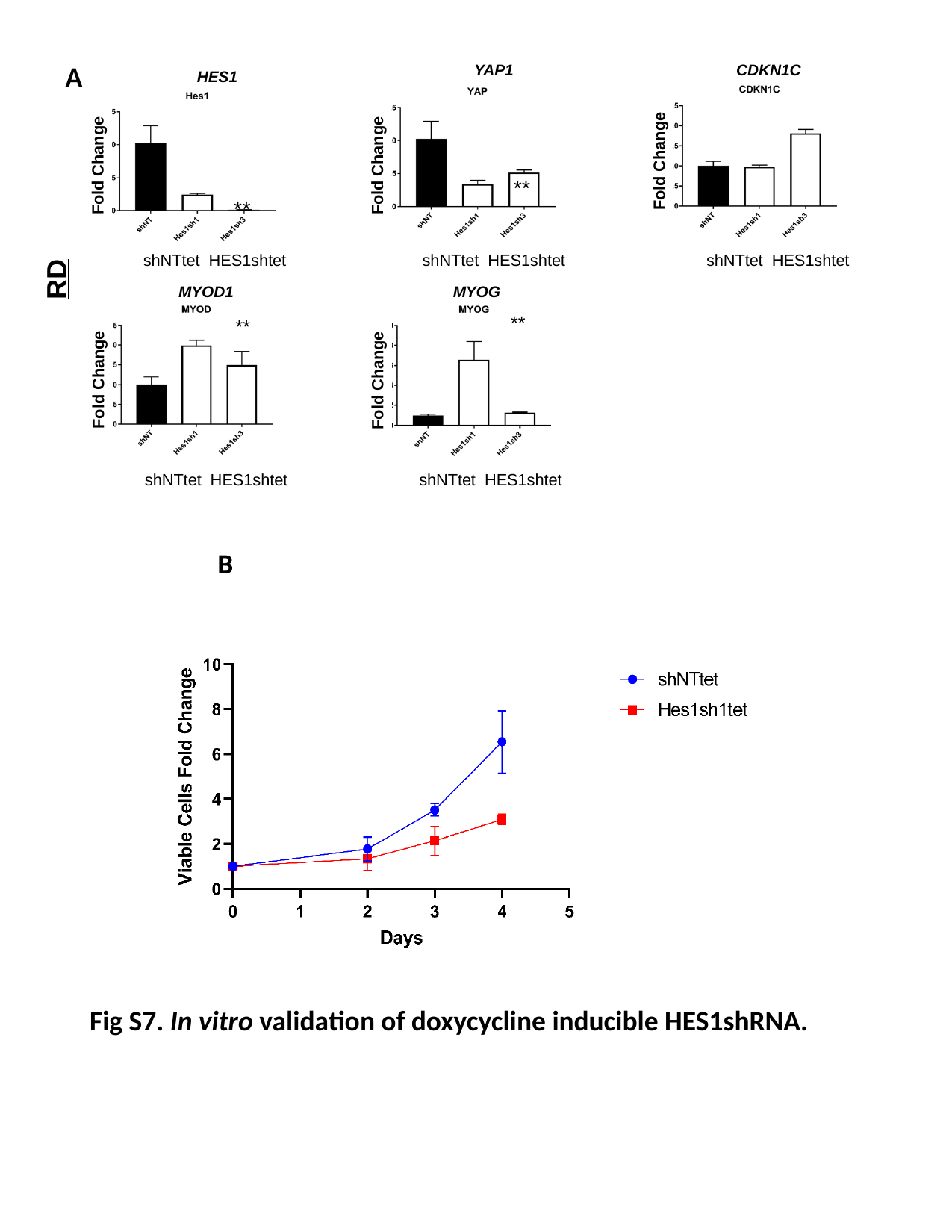

A
YAP1
CDKN1C
HES1
**
**
RD
MYOG
MYOD1
**
Fold Change
Fold Change
Fold Change
shNTtet  HES1shtet
shNTtet  HES1shtet
shNTtet  HES1shtet
**
Fold Change
Fold Change
shNTtet  HES1shtet
shNTtet  HES1shtet
B
Fig S7. In vitro validation of doxycycline inducible HES1shRNA.

## Slide 9
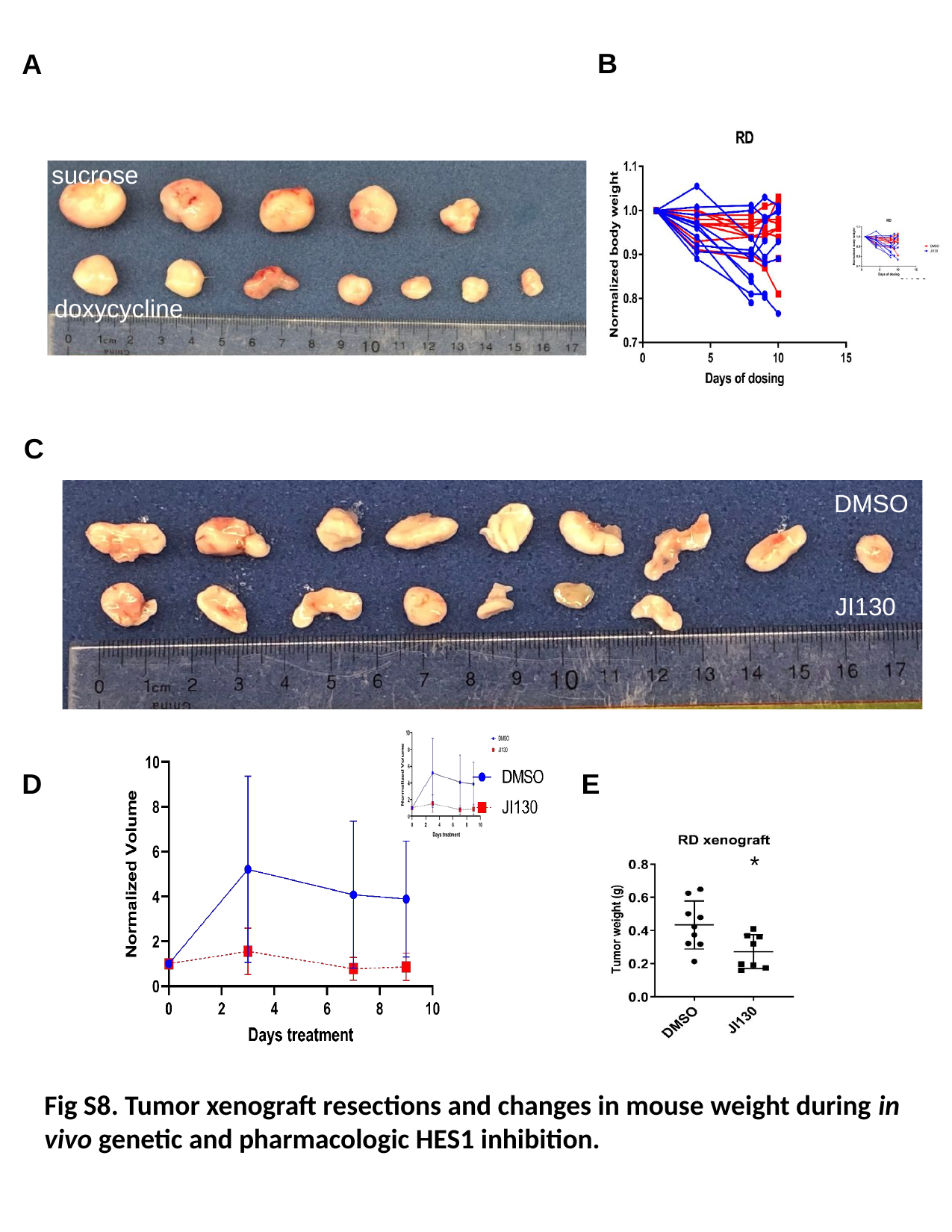

B
A
sucrose
doxycycline
C
DMSO
JI130
D
E
*
Fig S8. Tumor xenograft resections and changes in mouse weight during in vivo genetic and pharmacologic HES1 inhibition.

## Slide 10
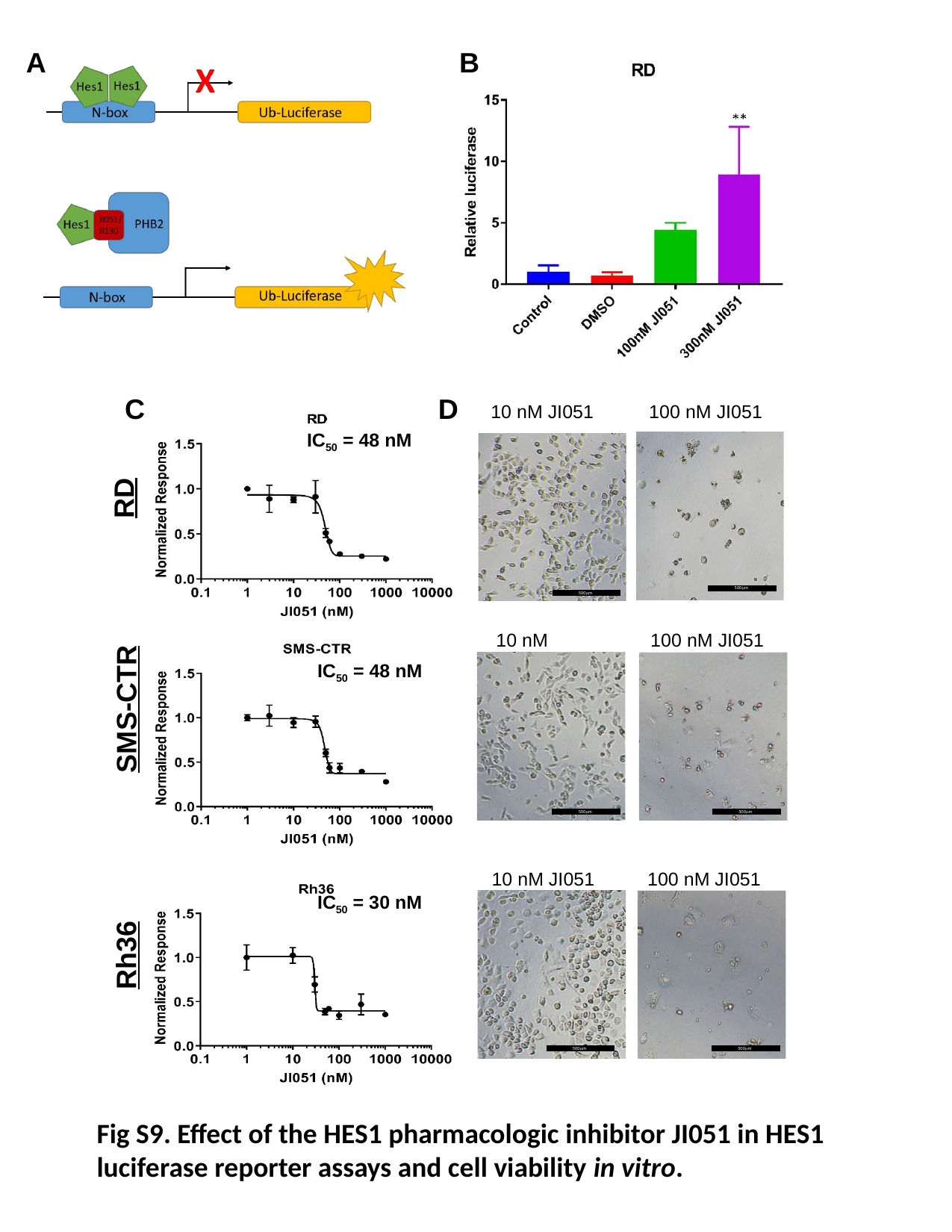

A
B
C
D
100 nM JI051
10 nM JI051
IC50 = 48 nM
500μm
500μm
RD
100 nM JI051
10 nM JI051
IC50 = 48 nM
SMS-CTR
500μm
500μm
10 nM JI051
100 nM JI051
IC50 = 30 nM
Rh36
500μm
500μm
Fig S9. Effect of the HES1 pharmacologic inhibitor JI051 in HES1 luciferase reporter assays and cell viability in vitro.

## Slide 11
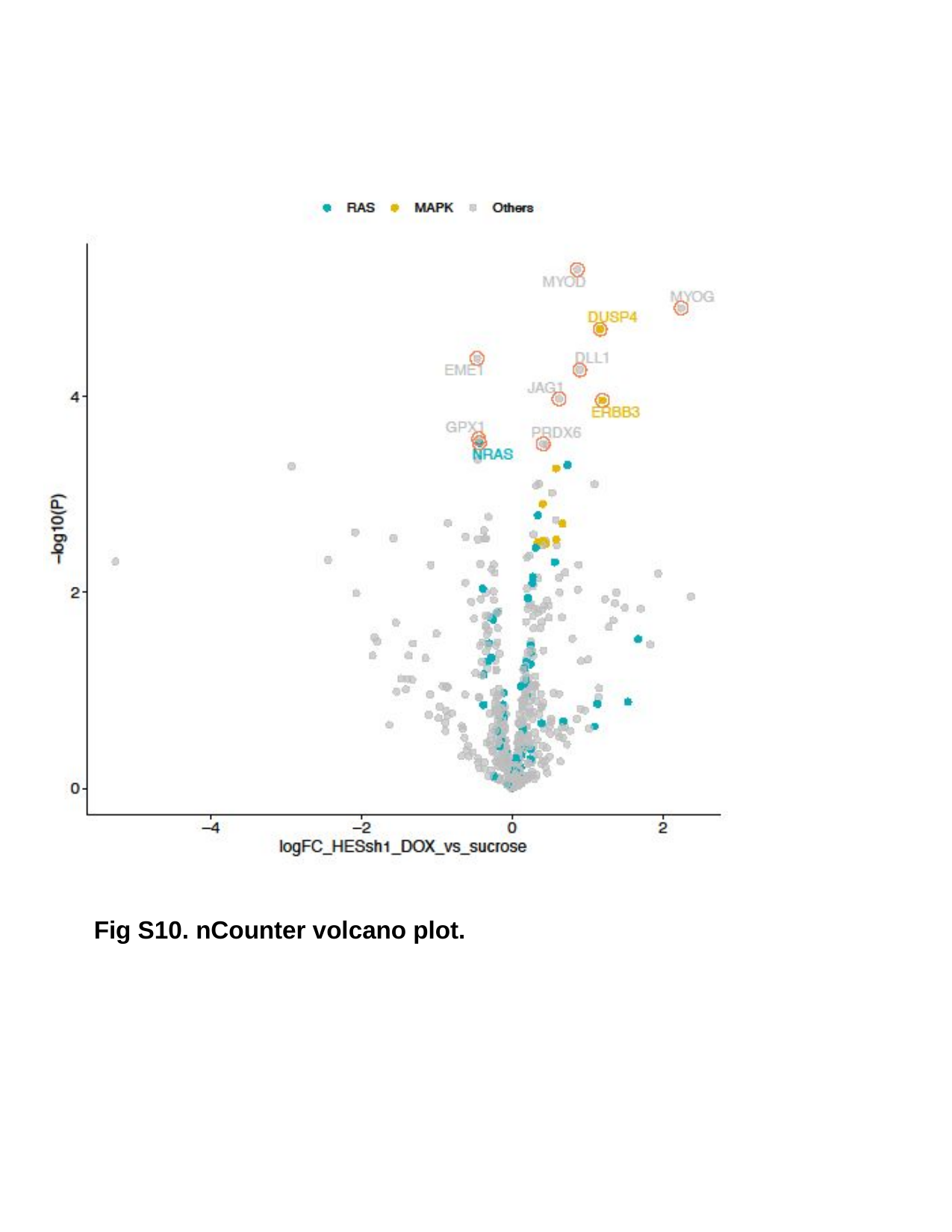

Fig S10. nCounter volcano plot.

## Slide 12
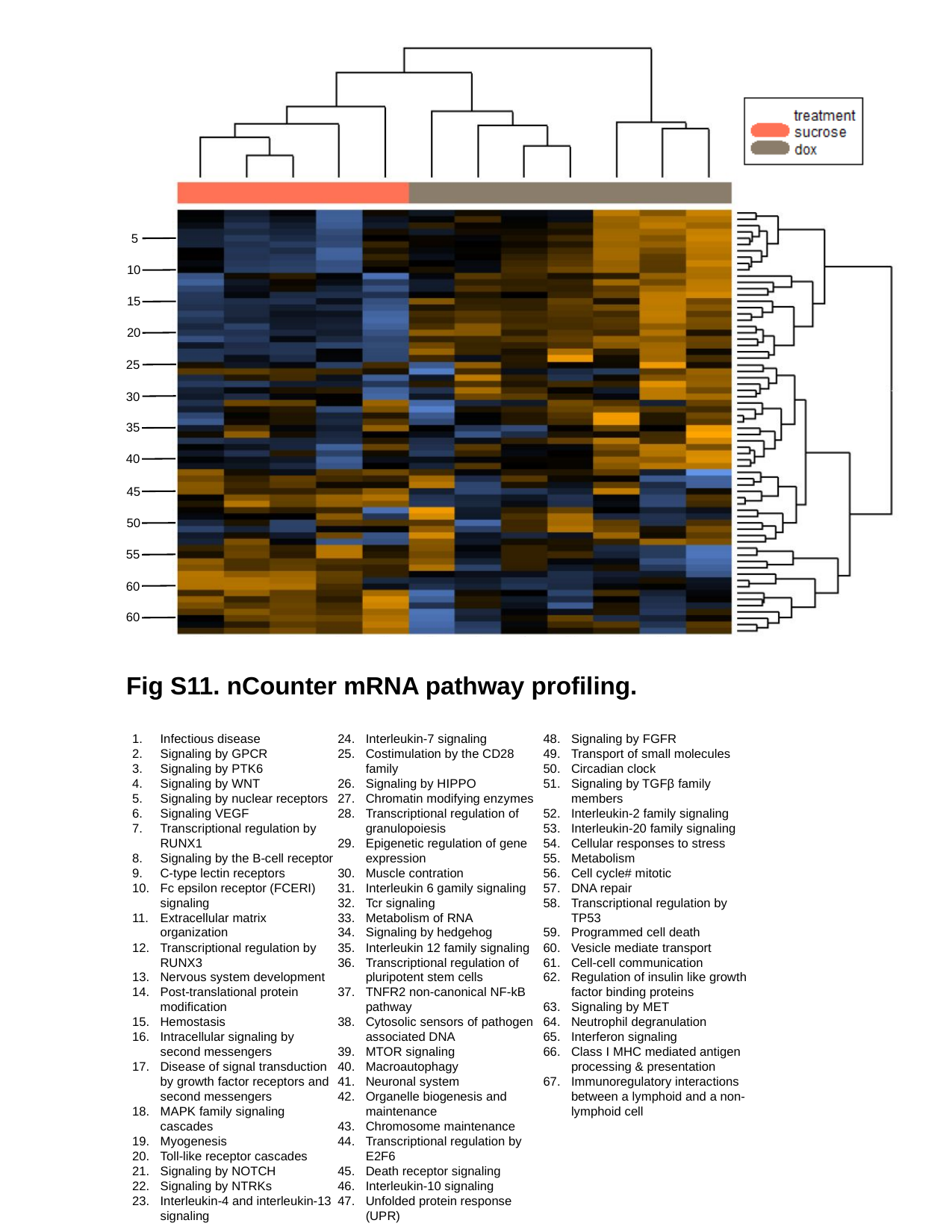

5
10
15
20
25
30
35
40
45
50
55
60
60
Fig S11. nCounter mRNA pathway profiling.
Infectious disease
Signaling by GPCR
Signaling by PTK6
Signaling by WNT
Signaling by nuclear receptors
Signaling VEGF
Transcriptional regulation by RUNX1
Signaling by the B-cell receptor
C-type lectin receptors
Fc epsilon receptor (FCERI) signaling
Extracellular matrix organization
Transcriptional regulation by RUNX3
Nervous system development
Post-translational protein modification
Hemostasis
Intracellular signaling by second messengers
Disease of signal transduction by growth factor receptors and second messengers
MAPK family signaling cascades
Myogenesis
Toll-like receptor cascades
Signaling by NOTCH
Signaling by NTRKs
Interleukin-4 and interleukin-13 signaling
Interleukin-7 signaling
Costimulation by the CD28 family
Signaling by HIPPO
Chromatin modifying enzymes
Transcriptional regulation of granulopoiesis
Epigenetic regulation of gene expression
Muscle contration
Interleukin 6 gamily signaling
Tcr signaling
Metabolism of RNA
Signaling by hedgehog
Interleukin 12 family signaling
Transcriptional regulation of pluripotent stem cells
TNFR2 non-canonical NF-kB pathway
Cytosolic sensors of pathogen associated DNA
MTOR signaling
Macroautophagy
Neuronal system
Organelle biogenesis and maintenance
Chromosome maintenance
Transcriptional regulation by E2F6
Death receptor signaling
Interleukin-10 signaling
Unfolded protein response (UPR)
Signaling by FGFR
Transport of small molecules
Circadian clock
Signaling by TGFβ family members
Interleukin-2 family signaling
Interleukin-20 family signaling
Cellular responses to stress
Metabolism
Cell cycle# mitotic
DNA repair
Transcriptional regulation by TP53
Programmed cell death
Vesicle mediate transport
Cell-cell communication
Regulation of insulin like growth factor binding proteins
Signaling by MET
Neutrophil degranulation
Interferon signaling
Class I MHC mediated antigen processing & presentation
Immunoregulatory interactions between a lymphoid and a non-lymphoid cell

## Slide 13
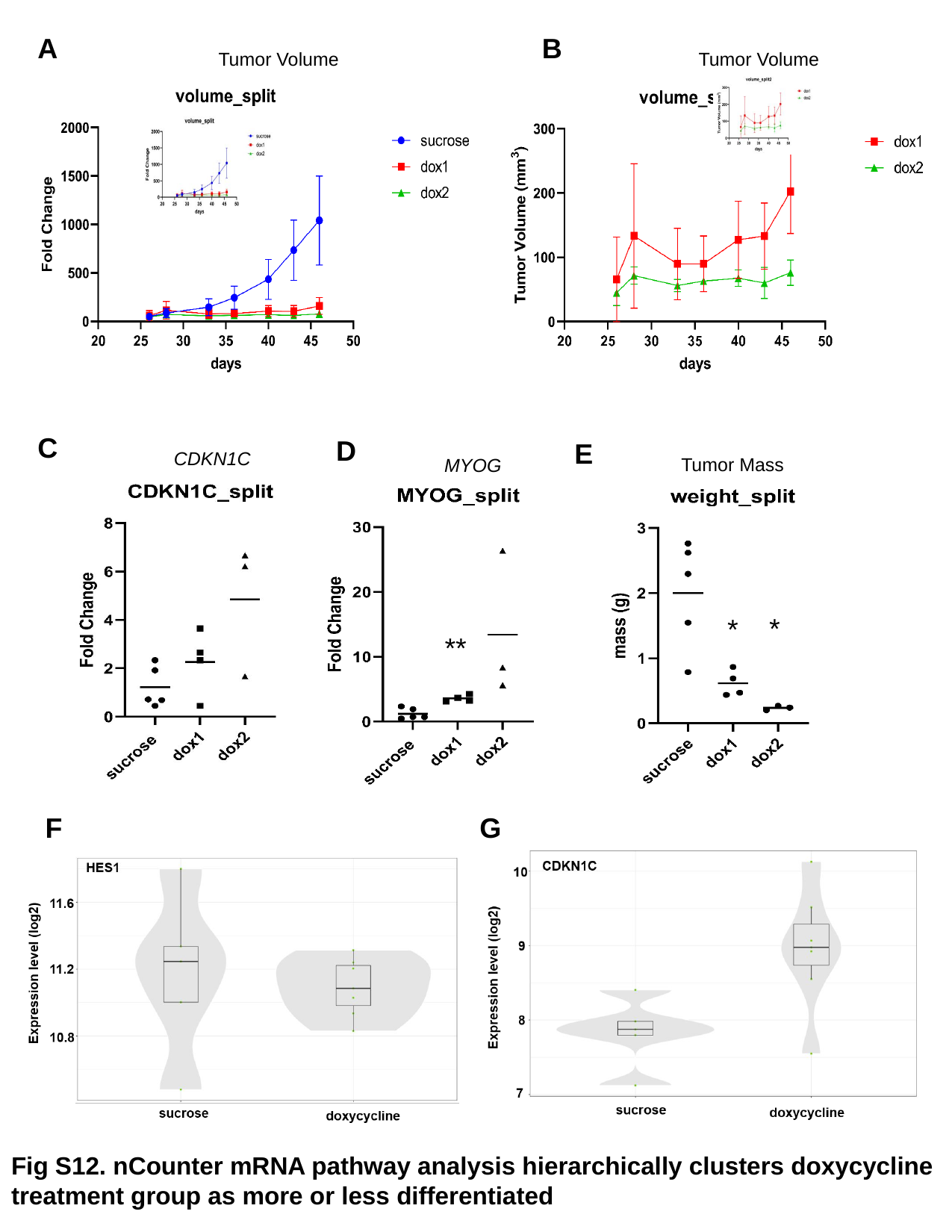

A
B
Tumor Volume
Tumor Volume
C
D
E
CDKN1C
Tumor Mass
*
*
MYOG
**
G
F
Fig S12. nCounter mRNA pathway analysis hierarchically clusters doxycycline treatment group as more or less differentiated

## Slide 14
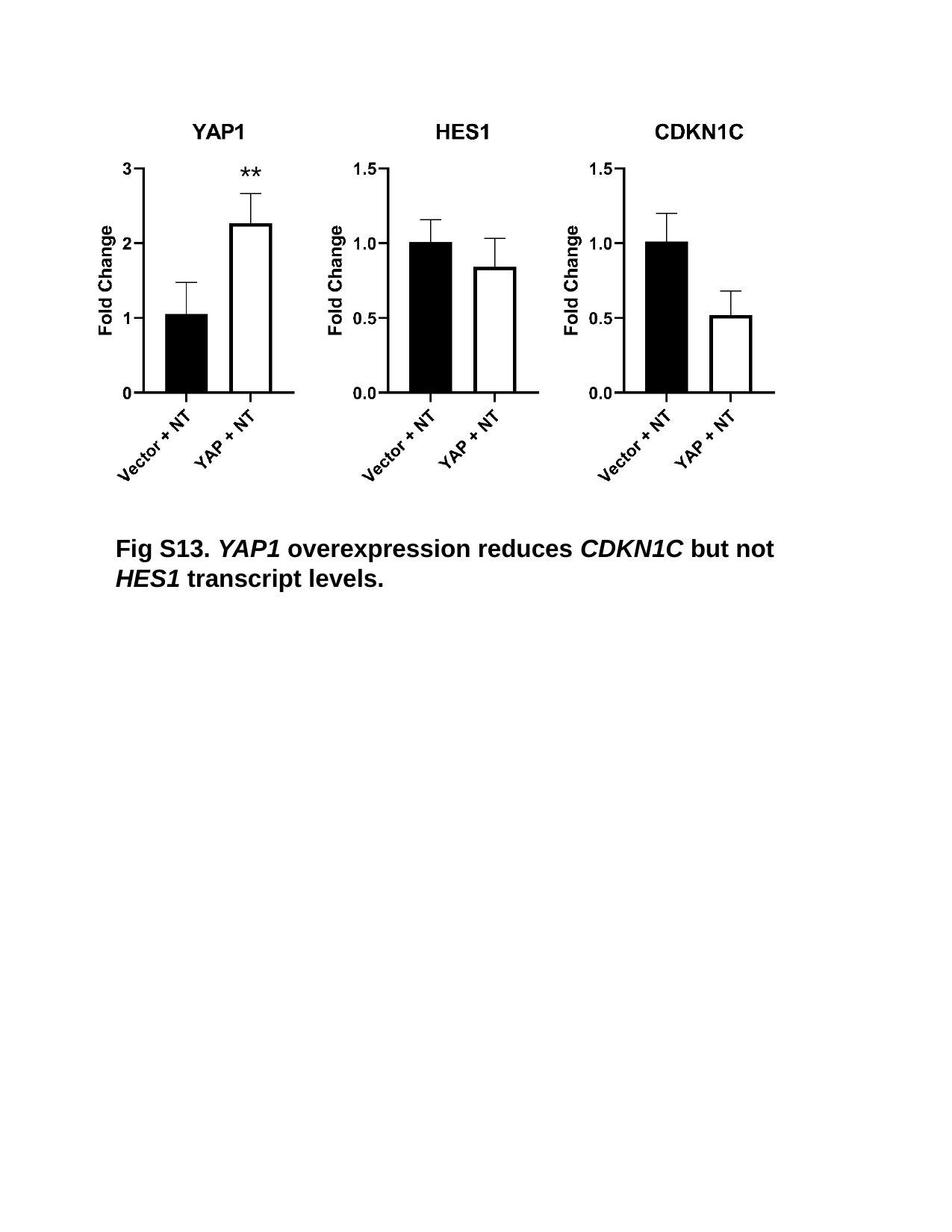

**
Fig S13. YAP1 overexpression reduces CDKN1C but not HES1 transcript levels.
