# Supplementary material for: Identification and targeting of a HES1‐YAP1‐CDKN1C functional interaction in fusion‐negative rhabdomyosarcoma
Source: Mol Oncol. 2022 Aug 29;16(20):3587–605. doi: 10.1002/1878-0261.13304 (PMC9580881; doi:10.1002/1878-0261.13304)
Supplement: Supplementary file 2 — Table S1. RT‐qPCR primers. [file MOL2-16-3587-s003.pdf]

Supplemental Table 1: RT-qPCR primers

| Gene | For | Rev |
|---|---|---|
| RPL32 | GGAGCGACTGCTACGGAAG | GATACTGTCCAAAAGGCTGGAA |
| B2M | GAGGCTATCCAGCGTACTCCA | CGGCAGGCATACTCATCTTTT |
| HES1 | TCAACACGACACCGGATAAAC | GCCGCGAGCTATCTTTCTTCA |
| YAP1 | CCTTCTTCAAGCCGCCGGAG | CAGTGTCCCAGGAGAAACAGC |
| CDKN1C | AGAGATCAGCGCCTGAGAAG | GGGCTCTTTGGGCTCTAAAC |
| WWTR1 | GGCTGGGAGATGACCTTCAC | CTGAGTGGGGTGGTTCTGCT |
| MYOD1 | GGTCCCTCGCGCCCAAAAGAT | CAGTTCTCCCGCCTCTCCTAC |
| MYOG | CAGTGCACTGGAGTTCAGCG | TTCATCTGGGAAGGCCACAGA |
| MRF4 | CCCCTTCAGCTACAGACCCAA | CCCCCTGGAATGATCGGAAAC |
