# Supplementary material for: Identification and targeting of a HES1‐YAP1‐CDKN1C functional interaction in fusion‐negative rhabdomyosarcoma
Source: Mol Oncol. 2022 Aug 29;16(20):3587–605. doi: 10.1002/1878-0261.13304 (PMC9580881; doi:10.1002/1878-0261.13304)
Supplement: Supplementary file 3 — Table S2. IHC antibodies. [file MOL2-16-3587-s002.pdf]

Supplemental Table S2: IHC antibodies

Antibody	Vendor - Cat	Dilution
HES1	Santa Cruz - sc-166410	1:200
Ki67	Abcam - ab16667	1:200
MYOD1	Dako - M3512	1:200
MYOG	Dako - M3559	1:200
