# Supplementary material for: Identification and targeting of a HES1‐YAP1‐CDKN1C functional interaction in fusion‐negative rhabdomyosarcoma
Source: Mol Oncol. 2022 Aug 29;16(20):3587–605. doi: 10.1002/1878-0261.13304 (PMC9580881; doi:10.1002/1878-0261.13304)
Supplement: Supplementary file 5 [file MOL2-16-3587-s001.docx]

**Identification and targeting of a HES1-YAP1-CDKN1C functional interaction in fusion-negative rhabdomyosarcoma**

Alexander R Kovach^1*^, Kristianne M Oristian^2,3*^, David G Kirsch^2,3^, Rex C Bentley^4^, Changde Cheng^5^, Xiang Chen^5^, Po-Han Chen^6^, Jen-Tsan Ashley Chi^6^, Corinne M Linardic^1,2^

^1^Department of Pediatrics, Duke University School of Medicine, Durham, NC, USA

^2^Department of Pharmacology & Cancer Biology, Duke University School of Medicine, Durham, NC, USA

^3^Department of Radiation Oncology, Duke University School of Medicine, Durham, NC, USA

^4^Department of Pathology, Duke University, Durham, NC USA

^5^Department of Computational Biology, St. Jude Children's Research Hospital, Memphis, TN, USA.

^6^Department of Molecular Genetics & Microbiology, Duke University School of Medicine, Durham, NC USA

*These authors contributed equally.

**Legends to supplemental figures**

___________________________________

Running title: HES1 in fusion-negative rhabdomyosarcoma

Key words: Rhabdomyosarcoma, HES1, YAP1, CDKN1C

*Corresponding author: Dr. Corinne M. Linardic, Division of Pediatric Hematology-Oncology, Department of Pediatrics, Duke University School of Medicine, Box 102382 DUMC, Durham, NC, 27710, USA. Tel/Fax +1.919.684.3401, +1.919.681.7950, Email:corinne.linardic@duke.edu

**Figure S1. YAP1 promotes *CDKN1C* downregulation, and assessment of *HES1* shRNA constructs. (A)** Transcriptional profiles from normal skeletal muscle (control), YAPS127A-induced ERMS tumors (Dox ON), and regressing tumors (Dox OFF) were compared to a set of 362 genes impacting senescence in mammalian cells (from Coppé et al and Kuilman and Peeper). Genes in bold represent senescence-associated secreted proteins. YAP S127A dataset obtained from GSE47198, Tremblay et al. **(B)** Ectopic expression of wild type *YAP1* (left) or constitutively active *YAP1* S127A (right) decreases *CDKN1C* mRNA levels. **(C)** Suppression of Hippo effectors *YAP1* (left) but not *WWTR1* (right) led to an increase in *CDKN1C* mRNA. **(D)** Of the three shRNAs generated to HES1, sh1 and sh3 showed the strongest knockdown of HES1 and were used in this manuscript.

**Figure S2. Full immunoblots corresponding to Figure 1.**

**Figure S3. Full immunoblots corresponding to Figure 2A (top) and Figure 2B (bottom).**

**Figure S4. HES1 knockdown inconsistently suppresses WWTR1 expression. HES1 suppression does not significantly induce differentiation of SMS-CTR cells under normal growth conditions.** While both RD **(A)** and SMS-CTR **(B)** cells show decreased *WWTR1* transcript levels in response to HES1 sh1 (left), knockdown is inconsistent with sh3 and at the protein level (right). **(C)** SMS-CTR cells cultured under growth conditions and expressing non-targeting (left) or shRNAs against HES1 (center and right) did not stain significantly with MF20 antibody. Images are 200x total magnification with scale bars representing 100μm.

**Figure S5. Full immunoblots corresponding to Supplemental Figure S4.**

**Figure S6. Full immunoblots corresponding to Figure 3A,B.**

**Figure S7. *In vitro* validation of doxycycline inducible HES1shRNA. (A)** Dox-Inducible HES1shRNA effectively targets and degrades *HES1* mRNA in RD cells and is associated with an expected decrease in *YAP1* and increase in *MYOD1* and *MYOG*. Unexpectedly, *CDKN1C* did not increase in this dox-inducible system, but did increase in the *in vivo* tumor xenograft studies (see Fig. 4C). **(B)** HES1sh1 inhibits cell growth**.**

**Figure S8. Tumor xenograft resections and changes in mouse weight during *in vivo* genetic and pharmacologic HES1 inhibition. (A)** RD xenograft tumor resections from HES1 dox-inducible sh1 study from Fig. 4. **(B)** Mouse weight over time for JI130 study. **(C)** RD xenograft tumor resections from JI130 pharmacologic study. **(D)** Average tumor volume over time for JI130 pharmacologic study. **(E)** Endpoint tumor weight of DMSO-treated and JI130-treated tumors.

**Figure S9. Effect of the HES1 pharmacologic inhibitor JI051 in HES1 luciferase reporter assays and cell viability *in vitro.* (A)** When HES1 is active, it binds to the promoter of the reporter and inhibits luciferase expression. When HES1 is sequestered by JI051, the reporter is expressed. **(B)** RD cells show a dose-dependent induction of luciferase when treated with JI051. **(C)** JI051 inhibits growth in three FN-RMS cell lines RD, SMS-CTR and Rh36 with IC50 values in the low nanomolar range (values shown in insets.) **(D)** JI051 treated cells are sparser and appear more rounded compared to DMSO control. Images are 50x total magnification with scale bars representing 500μm.

**Figure S10. nCounter volcano plot.** nCounter confirms differential expression of *MYOD1* and *MYOG* mRNA transcripts in dox-inducible HES1sh1-expressing tumor xenografts and reveals alteration to MAPK (yellow) and other Ras pathway (blue) associated genes. logFC indicates log fold change of transcripts in the xenograft tumors harvested from doxycycline treated mice compared to those harvested from sucrose treated mice.

**Figure S11. nCounter mRNA pathway profiling.** mRNA profiling of the dox-inducible HES1sh1-expressing tumor xenografts using the Nanostring nCounter® platform and Tumor Signaling 360™ panel shows upregulated signaling pathways in gold, and downregulated pathways in blue. Sucrose-treated control tumors cluster to the left under the orange bar, whereas doxycycline-treated tumors are on the right underneath the gray bar, and are clustered into two distinct groups.

**Figure S12. nCounter mRNA pathway analysis hierarchically clusters doxycycline treatment group as more or less differentiated.** Doxycycline treated tumors expressing HES1sh1 hierarchically clustered by nCounter pathway analysis can also be grouped on the basis of **(A,B)** volume, **(C)** CDKN1C expression as measured by RT-qPCR, **(D)** MYOG expression as measured by RT-qPCR, and **(E)** endpoint tumor mass in grams**.** Increased differentiation is associated with smaller tumors. Violin plot with overlaid box plots depicting log2 transcript levels of **(F)** *HES1*, and **(G)** *CDKN1*C in tumor xenografts from the HES1 dox-inducible genetic suppression study as measured by the nCounter platform.

**Figure S13. *YAP1* overexpression reduces *CDKN1C* but not *HES1* transcript levels.** RT-qPCR analysis of RD cells expressing empty vector or constitutively active YAP S127A. Both cell lines also contain a scrambled non-targeting (NT) shRNA. **(A)** *YAP1* message is confirmed to be increased, whereas **(B)** *HES1* transcript is unchanged and **(C)** *CDKN1C* is decreased.
